# The Kainic Acid Models of Temporal Lobe Epilepsy

**DOI:** 10.1523/ENEURO.0337-20.2021

**Published:** 2021-04-08

**Authors:** Evgeniia Rusina, Christophe Bernard, Adam Williamson

**Affiliations:** 1 Institute de Neurosciences des Systèmes, Institut National de la Santé et de la Recherche Médicale, Unité Mixte de Recherche 1106, Aix Marseille Univ, Inserm, INS, Institut de Neurosciences des Systèmes, Marseille, France; 2 Laboratory of Organic Electronics, Campus Norrköping, Linköping University, Norrköping 602 21, Sweden

**Keywords:** kainic acid, hippocampus, mice models of temporal lobe epilepsy, EEG

## Abstract

Experimental models of epilepsy are useful to identify potential mechanisms of epileptogenesis, seizure genesis, comorbidities, and treatment efficacy. The kainic acid (KA) model is one of the most commonly used. Several modes of administration of KA exist, each producing different effects in a strain-, species-, gender-, and age-dependent manner. In this review, we discuss the advantages and limitations of the various forms of KA administration (systemic, intrahippocampal, and intranasal), as well as the histologic, electrophysiological, and behavioral outcomes in different strains and species. We attempt a personal perspective and discuss areas where work is needed. The diversity of KA models and their outcomes offers researchers a rich palette of phenotypes, which may be relevant to specific traits found in patients with temporal lobe epilepsy.

## Significance Statement

This review aims to help researchers use a knowledge-based approach to study specific aspects of human epilepsy phenotypes. We focus on the kainic acid (KA) model of temporal lobe epilepsy in rodents, presenting it as a set of sub-models, describing the various administration routes, and the differences in outcome between species, strain, age, and sex. We have reviewed >200 research articles, summarizing the data with a ready-to-use structure.

## Introduction

Mesial temporal lobe epilepsy (MTLE) is the most common type of partial epilepsy in adults (Engel, 2001). It is characterized by recurrent spontaneous seizures, often resistant to drug treatments (Engel et al., 2012). Numerous alterations have been reported in patients with MTLE, including hippocampal sclerosis (HS) and cell death (Berkovic et al., 1991), mossy fiber sprouting (Sutula et al., 1989), reorganization of hippocampal interneuronal networks (Sloviter et al.,1991; Muñoz et al., 2004; Tóth et al., 2010), alterations in neuropeptide signaling (Vezzani and Sperk, 2004; Kharlamov et al., 2007) and synaptic transmission regulation (Casillas-Espinosa et al., 2012), granular cell dispersion and gliosis (Blümcke et al., 2002), and blood-brain-barrier dysfunction and angiogenesis (Rigau et al., 2007).

A striking feature of MTLE in humans is its heterogeneity. There are various forms of HS in patients, which led to a consensus classification based on histopathological differences (Blümcke et al., 2013). Likewise, there is a large diversity in terms of semiologic and electrophysiologic features in temporal lobe seizures, including regional epileptogenicity and whether or not seizures secondarily generalize (Maillard et al., 2004; Barba et al., 2007; Bartolomei et al., 2008). A large variability also exists in hypometabolism in patients with TLE (Guedj et al., 2015). Finally, the diversity between patients is even more important when considering comorbidities (i.e., conditions co-occurring with seizures), such as cognitive deficits, anxiety, and depression (Holmes, 2015; de Barros Lourenço et al., 2020; Krishnan, 2020).

These considerations provide the context of this review. We need a wide array of phenotypes in experimental models to ask specific questions that may be relevant to subsets of patients. It is reasonable to propose that genetic and epigenetic differences strongly contribute to phenotypic diversity in patients. In contrast, basic research uses inbred rodents living in a “stable” environment in animal facilities. This facilitates the task of researchers, increasing the reproducibility of the results. However, a given model may only be relevant to a specific phenotype in patients. Fortunately, several experimental models of epilepsy have been developed, originally to mimic MTLE, including the pilocarpine models, the electrode-based kindling models, and the kainic acid (KA) models. Although homology between a rodent model and human epilepsy cannot be claimed, experimental models must reproduce the main symptom: spontaneous recurrent seizures (SRSs). If other pathologic traits (e.g., mossy fiber sprouting or HS) or comorbidities (e.g., cognitive deficits and depression) exist, it may be possible to achieve a degree of granularity compatible with the diversity of phenotypes found in patients. This review will focus on the KA rodent model, its advantages and limitations, methods of administration, histologic, electrophysiological, and behavioral outcomes. As we will see, this model (Table 1) alone is characterized by a wide range of phenotypes.

The ideal situation would be to map the diversity of KA phenotypes with that of MTLE phenotypes. Unfortunately, most of the time, the existing literature does not provide enough information. For example, the part of the hippocampus that is resected in patients corresponds to the ventral hippocampus in rodents. A minority of experimental studies (apart from intra/supra hippocampal injections) distinguish the ventral from the dorsal hippocampus, despite the fact that clear differences appear in experimental epilepsy when specifically studied (Bernard et al., 2007; Ekstrand et al., 2011; Isaeva et al., 2015; Evans and Dougherty, 2018; Arnold et al., 2019; Brancati et al., 2021; do Canto et al., 2020; Canto et al., 2021). Most electrophysiological recordings in rodent models are performed in the dorsal hippocampus, or supradurally, essentially capturing the activity of the dorsal hippocampus via volume conduction, not what happens in the ventral hippocampus. Likewise, morphologic studies rarely distinguish the ventral and dorsal hippocampi in terms of cell death, sprouting, etc. The use of common data elements in future studies will be particularly useful to try correlate experimental models and patient data.

The last cautionary note relates to the way rodent data are analyzed: results are averaged, which assumes that the model will produce similar results with some variation. The clinic teaches us otherwise. If we just consider the electrophysiological signature of seizures, patients with mesial temporal atrophy/sclerosis can display low-voltage fast (LVF) or hypersynchronous (HYP) seizures (Perucca et al., 2014; see their Table 1). Individual patients can display several types of seizures (Saggio et al., 2020). Rodents, even from the same litter, are not clones. They are also individuals (Gomez-Marin and Ghazanfar, 2019). They can react very differently to a given insult, including epileptogenesis, and produce different phenotypes (Becker et al., 2015, 2019; Medel-Matus et al., 2017). Most studies presented hereafter did not report major differences within experimental groups, but the data may be there. Looking for differences within a given batch of experimental animals may constitute a very promising line of research, in keeping with a highly clinically relevant question: the development of personalized medicine.

**Table 1:** Neuropathological alterations in different KA models in rats and mice

Administration mode	Neuropathology		References	
	Rats	Mice	Rats	Mice
Systemic	Bilateral damage Entire hippocampus Subiculum Entorhinal cortex MF sprouting	Bilateral damage CA3/CA1 Cortical areas Lateral amygdala Dorsal thalamus MF sprouting	Stafstrom et al. (1992) Lado (2006) Williams et al. (2009) White et al. (2010) Drexel et al. (2012) Bertoglio et al. (2017)	Hu et al. (1998) McKhann et al. (2003) Benkovic et al. (2004) Umpierre et al. (2016)
ICV	Unilateral lesion CA3/CA4 areas of the hippocampus MF sprouting	Unilateral damage CA3/CA1 areas of the hippocampus MF sprouting	Miyamoto et al. (1997) Jing et al. (2009) Gordon et al. (2014) Song et al. (2016) Gao et al. (2019)	Cho et al. (2002) Jeon et al. (2004) Park et al. (2008) Jin et al. (2009)
Intra-HC	Unilateral lesion CA3/CA4 DG granule cells dispersion MF sprouting	Unilateral lesion CA3/CA1 DG granule cells dispersion MF sprouting	Cavalheiro et al. (1982) Bragin et al. (2005) Arkhipov et al. (2008) Rattka et al. (2013) Klee et al. (2017)	Bouilleret et al. (1999) Riban et al. (2002) Gouder et al. (2003) Gröticke et al. (2008) Lee et al. (2012) Zeidler et al. (2018)
Intraamygdaloid	Ipsilateral Am Contralateral HC Contralateral Am CA3/CA1 MF sprouting Extratemporal	Ipsilateral Am Contralateral HC Contralateral Am CA3/CA1 MF sprouting Extratemporal	Ben-Ari et al. (1979) Tanaka et al. (1992) Ueda et al. (2001) Takebayashi et al. (2007) Gurbanova et al. (2008)	Araki et al. (2002) Shinoda et al. (2004) Mouri et al. (2008) Tanaka et al. (2010) Li et al. (2012) Liu et al. (2013)
Supra-HC	-	Unilateral lesion CA3/CA1 DG granule cells dispersion	-	Bedner et al. (2015) Jefferys et al. (2016) Pitsch et al. (2019)
Intranasal	-	Bilateral damage CA3 area of the hippocampus Olfactory bulbs	-	Chen et al. (2002) Duan et al. (2006) Zhang et al. (2008) Lu et al. (2008) Sabilallah et al. (2016)

Am, amygdala; DG, dentate gyrus; HC, hippocampus; ICV, intraventricular; MF, mossy fibers

Summarizing the data over the past decades is not an easy task. There was no standardized way to report experimental protocol and analysis methods, which prevents a real comparison between labs. Although 24-h video/EEG recordings are now routinely used in experimental rodent models, there were not as widespread in the past. Notwithstanding their importance, some studies used short recording sessions (a few hours per day, during working days) and could potentially have missed some epileptic activity. Some studies only used visual observation.

As mentioned previously, there is still no agreement between scientists on how to define a seizure in a rodent. While the Racine scale provides an attempt to organize and quantify behavioral seizures, subclinical seizures, frequent spikes, and bursts may be present on the EEG without overt behavioral correlates. Considering the differences in seizure profile between rats and mice and the variability between labs, it is really hard to draw clear conclusions from the huge pool of data collected through so many years.

The problems of standardization also apply to neuroanatomical and functional morphologic studies. Many confounding factors can affect the interpretation of the observations. The counting method is important. Stereological techniques were not available in the past. Hence it is difficult to compare studies even when the same experimental model of epilepsy is used. Furthermore, the hippocampus is heterogeneous in terms of structure, cell distribution, and properties along both septo-temporal and longitudinal axes, and even within the pyramidal cell layer (Sotiriou et al., 2005; Pandis et al., 2006; Thompson et al., 2008; Dong et al., 2009a; Mizuseki et al., 2011; Malik et al., 2016; Sharif et al., 2021).

It is not always clear which regions/subfields are being investigated, e.g., to assess cell death. Morphologic alterations may be regionalized. Human data demonstrate even more complex changes as basket innervation is patchy from one subfield to the next in a given patient (Arellano et al., 2004). Such heterogeneity is also found in multidrug transporters’ expression, which can be very different from one slice to the next obtained after neurosurgery (Sandow et al., 2015). In animal models, we tend to assume that neuroanatomical changes are homogeneous, i.e., that the measurements performed in a few sections can be generalized to the structure. Many other brain regions are also characterized by spatial gradients, which should be considered when assessing circuit alterations. Likewise, since it is cumbersome to do, few studies engage in a global assessment of anatomic alterations in different regions in experimental models; much work is needed to address this. It would be helpful to use common data elements to standardize protocols and facilitate comparisons between studies (Lapinlampi et al., 2017).

We have attempted to be as neutral as possible in our evaluation, relying on the interpretation provided in each study and, whenever relevant, providing our own experience. In the following, we describe the different phenotypes found in various KA models. Given the wide heterogeneity found in patients, all models described hereafter reproduce some features found in some patients. Most experimental models share similarities with properties found in a majority of MTLE patients, including electrophysiological (seizures, interictal spikes, high-frequency oscillations) and morphologic (granule cell dispersion, mossy fiber sprouting) signatures. We will not take a position regarding a possible relevance to features found in patients, because there is no prototypical signature of MTLE at the morpho-functional level. For example, HS is found in only 60% of MTLE cases (Thom, 2014), and patients with MTLE can have LVF or HYP seizures (Perucca et al., 2014). The choice of the model should be tailored to the scientific question, which is laboratory specific. We describe and provide tables listing the various observations made in different models. These tables can be used for knowledge-based approaches. For example, if one is interested in the mechanisms of LVF seizures in patients with end folium sclerosis, identifying an experimental model reproducing such features (if available), and studying it. We also highlight issues that need to be investigated, which could constitute interesting opportunities for young investigators.

## Models of TLE: A Brief Overview

The main goal of creating a reliable animal model of epilepsy is to develop a chronic condition, which is consistent and efficient in generating SRSs. Several methods have been developed, all of them are based on different approaches, but they generally use an initial brain insult. It is worth noting that despite the fact that all these methods produce reliable epilepsy models, they do not always show the same phenotypes, such as neuronal cell death, neuropathological alterations, and epileptogenesis. The reasons behind such heterogeneity are not clear. Every single model has its nuances, which should be seen as a strength. We provide a brief information about other TLE models to stress further the diversity that can be achieved in terms of phenotypes. We did not attempt to compare them to the kainate models, which was beyond this review’s scope. We are also not discussing genetic conditions and transgenic strains that exhibit a TLE-like phenotype (Toth et al., 1995; Zeng et al., 2011), but it is worth mentioning that TLE may have a genetic predisposition (Andermann et al., 2005). We do not want to convey the wrong impression that we favor the KA model. All models are interesting as they enable researchers to tap into a rich repertoire of phenotypes. Although this brief description of other than KA models does not do justice to them, we start this review with these models to highlight their diversity and richness.

The administration of various chemoconvulsants either systemically or directly into the brain is commonly used to trigger epileptogenesis, the process that leads to the development of SRSs. Pilocarpine and KA are broadly used chemoconvulsants. Pilocarpine, a muscarinic acetylcholine receptor agonist, was first proposed to generate experimental epilepsy in rats by Turski et al. (1983). Intraperitoneal injection of 100–400 mg/kg pilocarpine triggers seizures starting with motor, olfactory, and gustatory automatisms, which evolve into status epilepticus (SE). Histologic examination of the brains revealed specific alterations throughout the hippocampal formation, amygdala, thalamus, neocortex, olfactory cortex, and substantia nigra (Turski et al., 1983). The most remarkable difference between the pilocarpine and the KA models is the rapidity of the pilocarpine one. Neuronal damage is visible within 3 h after pilocarpine-induced SE, while in the case of KA, the brain lesion in the same areas is visible 8 h after SE induction (Covolan and Mello, 2000). Regarding the extension of cellular damage, both KA and pilocarpine models, injected systemically, produce an extensive extrahippocampal cell loss (Lévesque and Avoli, 2013). In mice, the pattern of cell loss is strain dependent (Schauwecker, 2012). It is important to stress that each lab used/uses different evaluation methods for cell counting and general damage assessment, making comparisons difficult.

Mortality in the pilocarpine model appears higher. Lithium treatment 24 h before the convulsant injection can significantly reduce mortality, leading to a different phenotype (Curia et al., 2008). Multiple reports indicate a strain difference in the pilocarpine model (McKhann et al., 2003; Curia et al., 2008; Inostroza et al., 2011; Lévesque and Avoli, 2013). For instance, cell damage and mortality rates are more significant in Long–Evans and Wistar rat strains than Sprague Dawley rats (Curia et al., 2008; Inostroza et al., 2011). The Sprague Dawley strain also demonstrates less prominent neuronal damage than Wistar rats (Inostroza et al., 2011). Overall, the Long–Evans strain is the most sensitive strain to pilocarpine, followed by Wistar, while the Sprague Dawley strain exhibits minimal sensitivity. The dose response to pilocarpine is similar in mice and rats; however, mice appear to be more sensitive and display higher mortality rates than rats (Curia et al., 2008). Also, mice treated with pilocarpine are more likely to develop SRSs than KA-treated ones (Lévesque and Avoli, 2013). Generally speaking, several parameters are involved in phenotype generation in both pilocarpine and KA models: administration route, dosage, duration and severity of the initial insult, environmental conditions, etc. Evidently, the characteristics of experimental animals play a crucial role as well. Species, strain, age, and sex create multiple variations in the models and will be thoroughly discussed later. As it is hard to navigate between so many factors, we have made an attempt to summarize existing data in the tables, demonstrating the full diversity of the KA models. The pilocarpine model, not any less complex, is not discussed in details in the current review, but the reader is welcome to read further literature about the model (Curia et al., 2008; Lévesque and Avoli, 2013).

It is important to note that the initial experiments performed with KA or pilocarpine showed high death rates. SE was left unchecked, and some drugs used to try to stop it had unwanted effects. Thus, it is difficult to compare the results obtained with these studies, as they may have generated phenotypes different from those described nowadays. More recent TLE models show lower levels of fatality in both mice and rats, in particular when SE is monitored with EEG recordings. In our hands, we noted that SE might start electrographically 5 min before behavioral manifestations, particularly in mice. We also noted that aberrant electrographic activity continues after the injection of the most optimal combination of drugs used to stop SE (Brandt et al., 2015), up to the next day following SE induction. Although the drugs have relaxant effects (no or few motor events), the aberrant activity continues, hence the necessity to monitor continuously with EEG recordings. Since SE triggers epileptogenesis, it is difficult to standardize its duration and severity, which could be a variability source within the same group of injected animals. Finally, we observed seasonal variability. In our hands, rodents seem to be more sensitive to pilocarpine and KA, with a greater death rate during the summer, another possible variability source.

In terms of behavioral performance, spatial memory seems to be affected significantly in the pilocarpine model. However, pilocarpine-treated animals show reduced anxiety levels compared with the KA-treated rats (Inostroza et al., 2011).

The injection of tetanus toxin into the hippocampus is also used to trigger epileptogenesis. The first mention of this procedure dates as early as the 19th century, demonstrating that intracerebral injection of the toxin causes seizures in experimental animals (Roux and Borrel, 1898). Unlike KA, tetanus toxin does not elicit SE after its injection but efficiently generates SRSs within 2–21 d postinjection, which usually ceases after a few weeks (Jefferys and Walker, 2006). A standard dose causes nearly 30% pyramidal cell loss in CA1 in the unilateral hippocampus and 10% at distant sites. Moreover, loss of the dendritic spines in CA3 pyramidal neurons is also reported (Benke and Swann, 2004).

The next common approach for modeling TLE in rodents includes various types of electrical stimulation. The most widely used model is the kindling model, in which an electrode is implanted into the brain, and a seizure focus is created by repeated electrical stimulation. The term “kindling” was first proposed by Goddard and colleagues (Goddard, 1967), where, after several experimental trials, they demonstrated that daily electrical stimulation leads to the development of an epileptic focus in the brain, creating generalized convulsions and permanent changes in brain tissue (Goddard et al., 1969). The kindling model has been extensively used targeting various regions such as the hippocampal formation (Kairiss et al., 1984; Palizvan et al., 2001), the piriform cortex (Löscher and Ebert, 1996), and the perirhinal cortex (McIntyre et al., 1993). The electrical stimulation model’s primary hallmark is the gradual development of an epileptic activity, as compared with the chemical administration, in which the initial brain insult leads to the development of chronic epilepsy after a latent period. The kindling model is sometimes described as a “functional” model because of the absence of gross morphologic damage, which is characteristic of chemical models (Morimoto et al., 2004) and a general failure to induce chronic SRS (Goddard, 1983). Thus, it is rather a model of epileptogenesis but not of epilepsy. A variation based on an optogenetic approach (rather than electric stimulation) called “optokindling” has been introduced recently (Cela et al., 2019). Although it was used to produce a model for neocortical epilepsy, the same technique may create a TLE model. The main advantage of this kindling model is the ability to trigger seizures on demand. At more advanced kindling stages, SRSs can occur (Wada et al., 1975).

Another way to obtain SRSs using the kindling approach is to trigger SE. Lothman and Bertram developed a model of continuous hippocampal stimulation to induce chronic epilepsy (Lothman et al., 1989). This model, named self-sustaining limbic SE (SSLSE), triggers SE and thus epileptogenesis leading to the occurrence of SRSs. A similar model was proposed by Mazarati et al. (1998), who suggested a brief 15- to 30-min perforant path stimulation (previously used as a kindling model; Sloviter, 1987), which led to continuous self-sustained SE and the development of SRSs.

Most chemical and electrical models use SE to trigger epileptogenesis. It is reasonable to propose that SE is the main determinant of epileptogenesis. However, causality has not been clearly established. This issue could be addressed with optogenetics, which may be used to try to stop SE soon after its onset.

One of TLE’s most common causes in patients is traumatic brain injury (TBI; Pitkänen and Mcintosh, 2006). Various TBI models have been developed, in particular, the fluid percussion injury model (D’Ambrosio et al., 2004). A single severe injury produced by fluid percussion injury device is sufficient to trigger epileptogenesis in rodents with some similarity with human TLE, including morphologic alterations such as neuronal loss in the hippocampus and mossy fiber sprouting (Kharatishvili et al., 2006; Pitkänen and Immonen, 2014).

The previous models involve brain insults triggered in adult animals. Insults occurring during development can also lead to TLE later in life. For example, febrile seizures are a risk factor for TLE development in humans (French et al., 1993). Hyperthermia-induced seizure models have been developed (Holtzman et al., 1981). Dubé et al. (2010) demonstrated that prolonged 70-min febrile seizures in postnatal day (P)11 rat pups could trigger epileptogenesis.

Neonatal hypoxia can also constitute an initial insult, resulting in TLE later in life (Jensen et al., 1991). It is linked to the observation that hypoxic encephalopathy is the most common cause of neonatal seizures (Aicardi and Chevrie, 1970). Exposing P10–P12 pups to graded global hypoxia (7−4% oxygen) for 15 min triggers epileptogenesis (Rakhade et al., 2011). Morphologic changes include mossy fiber sprouting, and electrophysiological analysis shows increased excitability, facilitated long-term potentiation induction, and longer after discharges.

The developing brain is not a miniature adult brain. Hence, specific models need to be developed to study epilepsies that occur during development. The same issue applies to the aging brain, for which mechanisms of epileptogenesis and seizure genesis may be specific to a given age. These are key questions, but they were beyond the scope of the present review.

There are various ways to trigger epileptogenesis in the brain, each leading to slightly different phenotypes. We will now focus on the KA model.

## The KA Model

KA, a cyclic analog of L-glutamate and an agonist of the ionotropic KA receptors (KARs), was first reported to damage hippocampal pyramidal neurons by Nadler et al. (1978). However, the use of KA as a model for epilepsy was first introduced by Ben-Ari and colleagues (Ben-Ari and Lagowska, 1978; Ben-Ari et al., 1979), who performed a unilateral intra-amygdaloid injection of KA in unanaesthetized non-paralyzed rats and observed focal seizures evolving into SE as the dosage increased. Moreover, the histologic findings revealed neuronal degeneration and gliosis in the CA3 field of the hippocampus. These, and other experiments, suggested using KA as a tool to model TLE in rodents. The injection of KA will lead to the activation of its cognate receptors.

### KARs

Extensive biomolecular research has provided us with information about the localization of KARs in the mammalian brain. Experiments on KAR mapping showed that these receptors could be found at different levels of expression throughout the brain, including the entorhinal cortex (Patel et al., 1986), cerebellum (Wisden and Seeburg, 1993), amygdala (Rogawski et al., 2003), basal ganglia (Jin and Smith, 2011), and the hippocampus in which they are particularly abundant (Bloss and Hunter, 2010). KARs belong to a family of ionotropic glutamate receptors, along with AMPA and NMDA receptors. They can be presynaptic or postsynaptic. Presynaptic KARs act bidirectionally, performing an excitatory action through their ionotropic activity and inhibition via a “non-canonical” metabotropic signaling (Lerma and Marques, 2013). Postsynaptic receptors contribute to excitatory neurotransmission (Huettner, 2003). KARs can also control GABAergic neurotransmission, both presynaptically and postsynaptically (Cossart et al., 2001a,b). There are five known types of KAR subunits: GluR_5_ (GluK1), GluR_6_ (GluK2), GluR_7_ (GluK3), KA1 (GluK4), and KA2 (GluK5). Some subunits are highly expressed in the hippocampus. The GluK4 subunit is almost exclusively found in the CA3 hippocampal field, whereas its expression in CA1 is limited (Bahn et al., 1994; Darstein et al., 2003). GluK5 subunits are expressed in both CA1 and CA3 fields (Bahn et al., 1994) and in the cortex and striatum (Gallyas et al., 2003). The fact that these two subunits have a high affinity for KA (dissociation constant of 5–15 nm) and are mainly concentrated in CA3 may explain the pattern of excitotoxic damage found in this region (Fernandes et al., 2009) and suggests that they are likely responsible for neuronal cell death.

GluK1 are found in the hippocampus’ CA3 field (Bahn et al., 1994), and GluK2 are highly expressed in both CA1 and CA3 (Bloss and Hunter, 2010). Although both subunits have low affinity for KA (K_D_ of 50–100 nm), GluK1 knock-out mice show increased susceptibility to the epileptogenic effect of KA, while GluK2 ablation prevents the generation of epileptiform discharges (Fisahn et al., 2004). GluK2-KO mice are also less sensitive to KA’s epileptogenic effect (Mulle et al., 1998). Conversely, overexpression of GluK2 by HSVGluR6 viral vector injection leads to seizure induction and hyper excitability (Telfeian et al., 2000). Furthermore, Ullal et al. (2005) reported that the GluR7 subunit, which has the lowest affinity to glutamate, is downregulated by KA-induced seizures in the long-term. These findings suggest that the KA administration will target all types of kainate receptors, with a unique effect in the hippocampus. Interestingly, the GluK1 subunit has been associated with acute seizure induction (Fritsch et al., 2014), since the acute effect is believed to be mediated by regulation of inhibition and GluK1 subunits are especially abundant in hippocampal interneurons (Paternain et al., 2000). Chronic epilepsy, however, likely develops because of GluK2 and GluK5 subunits, which are found in the newly formed KARs within the mossy fiber network (Pinheiro et al., 2013; Artinian et al., 2015). Increased expression of GluK4 has been found in the brains of patients with refractory TLE (Lowry et al., 2013). Additionally, GluK4 knock-out mice demonstrated full neuroprotection in the CA3 area of the hippocampus following administration of KA (Das et al., 2012). Thus, it is reasonable to assume that GluK4 modulates KA-induced neurodegeneration.

It could be interesting to determine which KARs in which cells are the main triggers of epileptogenesis. The recent development of photoswitchable regulators of ligand-gated channels (Bregestovski et al., 2018) could enable a tight control of specific KARs in a cell type and brain region-dependent manner.

### Mechanism of action

**Figure 1.**
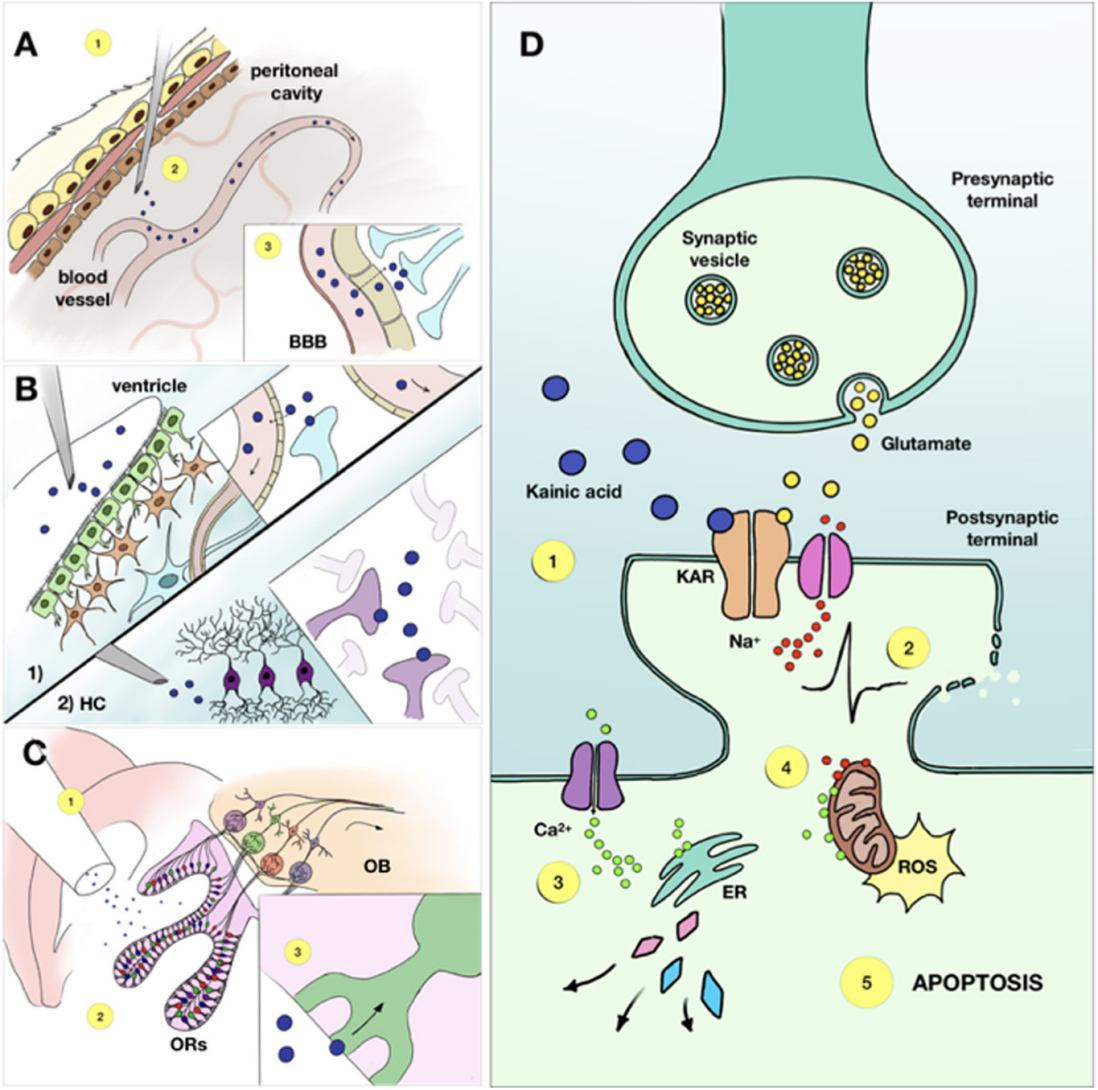
The complex mechanism of KA-induced neuronal damage includes a sequence of events and its outcome varies depending on the administration route. ***A***, The intraperitoneal route of KA administration is realized through an injection of the drug into the peritoneal cavity (1). The molecules then get absorbed by blood vessels (2) and travel to the brain, where they pass the blood-brain barrier via passive diffusion (3). ***B***, The intracerebral administration can be performed in various zones but the most common are intraventricular (1) and intrahippocampal (2). In case of intraventricular injection, the drug molecules diffuse directly into the cells surrounding the ventricle wall and another part is taken by the blood vessels to be distributed throughout the entire brain. Intrahippocampal administration, on the contrary, provides a more localised damage, as KA molecules activate KA receptors in the hippocampus at the site of injection. ***C***, The intranasal route starts with the injection of KA in the nasal cavity (1), where the molecules are absorbed by receptors of the olfactory epithelium (2), from where they travel through the olfactory pathway to the hippocampus and other areas of the brain (3). ***D***, KA, once having reached the brain tissue, initiates a cascade of events First, it binds to the KARs, causing membrane depolarization and cell firing (1). Excessive firing can lead to osmotic imbalance and, eventually, rupture of the postsynaptic membrane (2). At the same time, influx of calcium into the cell results in multiple enzymes activation, such as phospholipase, endonucleases and proteases, all of which damage various cell structures (3). Additional effect of an increased intracellular Ca2+ concentration is mitochondrial disfunction, and excessive production of reactive oxygen species (4). All these mechanisms potentiate each other and terminate in apoptosis (5). BBB — blood-brain barrier, ER — endoplasmatic reticulum, HC — hippocampus, KAR — kainic acid receptor, OB — olfactory bulb, ORs — olfactory receptors, ROS — reactive oxygen species.

Excitotoxicity refers to a process in which neurons experience severe damage to the point of cell death because of overstimulation by excitatory neurotransmitters such as glutamate (Dong et al., 2009b). The mechanism behind this includes a cascade of molecular interactions that lead to osmotic imbalance, excessive depolarization, and, eventually, rupture of the postsynaptic membrane (Beck et al., 2003). Several mechanisms are at play. A central one involves the intracellular accumulation of Ca^2+^, following the excessive activation of glutamate receptors (Fig. 1*D*). The rise in Ca^2+^ can strongly impact mitochondria and the endoplasmatic reticulum (Friedman, 2006). Elimination of intracellular Ca^2+^ or blocking its influx into mitochondria can diminish cellular sensitivity to apoptotic stimuli (Lee et al., 2009). The Na^+^ and Cl^−^ ions are also involved since their removal from the extracellular space stop neurodegeneration (Chen et al., 1998). Finally, extracellular K^+^ is also engaged in KA-induced excitotoxicity (Ha et al., 2002).

Oxidative stress also plays a central role in cell death in the context of excitotoxic damage. The excess of glutamate initiates reactive oxygen species (ROS) formation, which leads to mitochondrial dysfunction and molecular damage (Murphy et al., 1989; Nguyen et al., 2011). KA injection leads to high levels of ROS (Chuang et al., 2004; Gano et al., 2018), as seen in brain tissue from TLE patients (Rowley and Patel, 2013).

Although oxidative stress is often considered as the main mechanism of cell death in TLE, many authors argue that apoptotic cell death contributes to the SE-induced brain damage or even prevails. Wasterlain et al. (1993) summarized and explained all the pathophysiological mechanisms that are associated with SE. Apparently, gene changes induced by SE can lead to apoptotic neuronal death, and it is possibly tied to the excitotoxic damage component. Mitochondrial membranes, being a subject of oxidative stress, activate apoptotic-inducing factor (AIF), which gets translocated into the nuclei and initiates DNA fragmentation (Fujikawa, 2005). Thus, it can be proposed that excessive glutamate release triggered by KAR activation, leads to excitotoxic cellular damage, which, in turn, activates apoptotic factors, and results in both apoptotic and excitotoxic cell death. However, this aspect is still debated.

The mechanisms of KA-induced SE remain incompletely understood. However, in different species and strains, KA-induced SE may result in different phenotypic traits. More generally, we observe different phenotypes when we use different independent variables such as the mechanisms of SE induction (e.g., pilocarpine, TBI, KA, etc.), species, strain, gender, age, etc. Intuitively, the state of the brain circuits at the moment of the induction of SE will be central to the future outcome. Since we cannot have access to the brain’s state, it is not yet possible to explain why different phenotypes are obtained. At present, we can only observe and describe them. A full understanding would allow a knowledge-based development of epilepsy models with the desired phenotype. Much work is still needed to reach this stage. At present, our personal opinion is that the mechanisms of induction of epileptogenesis (how and where KA acts) are less important than the phenotypic traits that one wishes to study.

## Administration Routes

**Figure 2.**
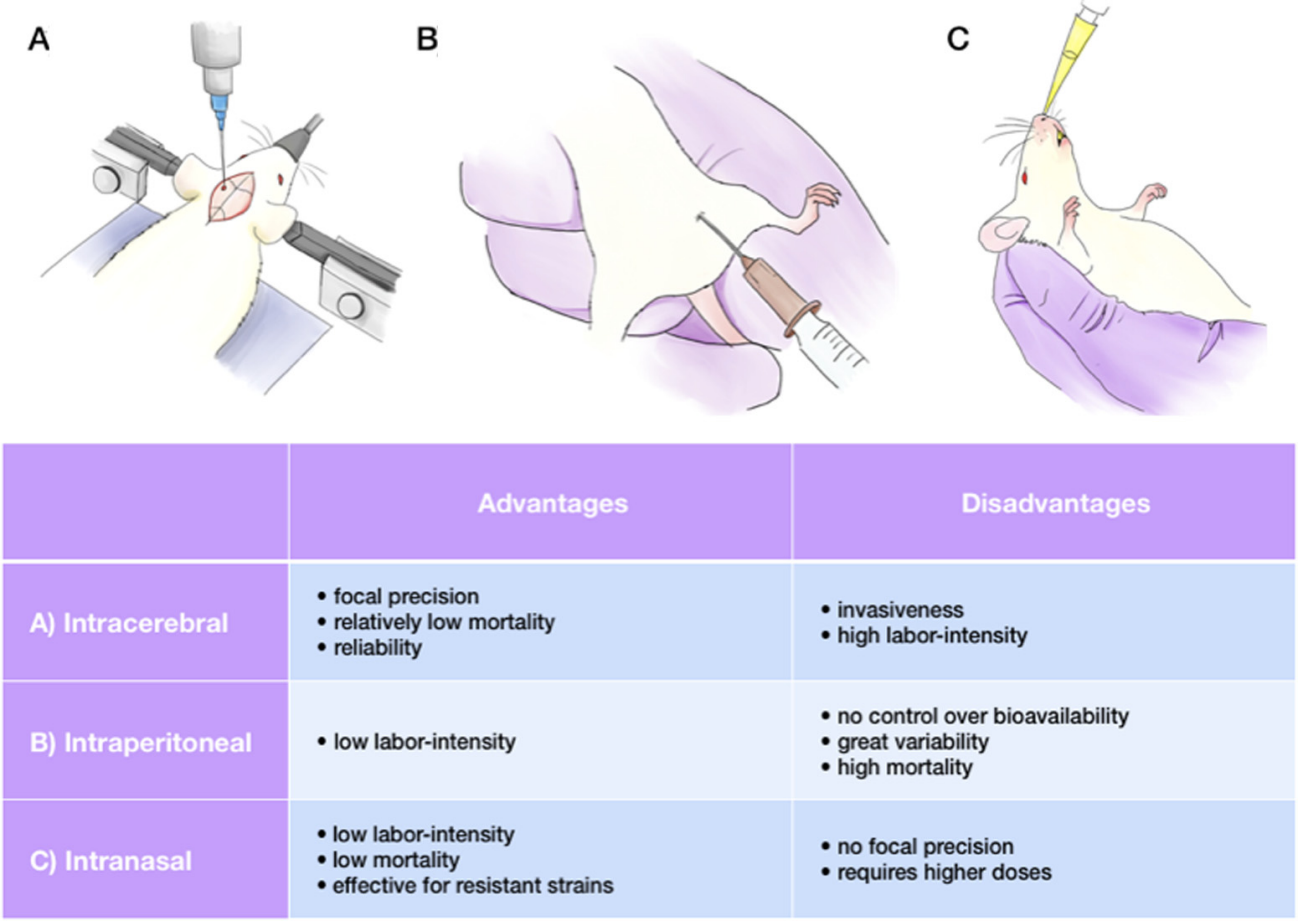
Advantages and disadvantages of KA administration routes. Various points should be taken into consideration, including age, sex and strain of an animal. ***A***, The main advantage of the intracerebral administration route is focal precision; this method is widely used, despite of its invasiveness and labor-intensity. ***B***, The intraperitoneal injection is easy to perform, but might result in high mortality, along with large outcome variability and uncontrolled tissue uptake. ***C***, The intranasal route leads to low mortality rates and works for resistant strains, but lacks focal precision.

KA, like many other drugs, can be administered in various ways, depending on the desired outcome (Figs. 1*A–C*, 2). Each method has its advantages and drawbacks and should be chosen accordingly. The key parameters one should consider selecting the injection route are mortality rate, labor-intensity, lesion control, and, finally, age, sex, and strain of the animal. The major administration routes are systemic, intracerebral (which could be divided into intraventricular, intrahippocampal, suprahippocampal, and intra-amygdaloid), and intranasal. Each of them is described further. The striking feature that will immediately appear is the diversity in terms of features, including the duration of the latent period, seizure properties and morpho-functional alterations, even within a given model. This should not be seen as blurring the picture. Rather it unravels the richness and diversity of all these models. The tables listing the different features as a function of model, sex, strain, etc. are provided to help researchers choosing the model best fitting their questions.

### Systemic administration

The first experiments using systemic KA administration were performed in the late 1970s to determine whether the toxin could induce damage via axonal connections (Schwob et al., 1980; Fig. 1*A*). The studies showed that rats injected by 12 mg/kg of KA intraperitoneally experienced the onset of “wet dog shaking” seizures ∼30–90 min after injection, which eventually evolved into secondary generalized tonic-clonic convulsions in 88% of cases. Neuronal damage was present already at 3 h postinjection and gradually increased for two weeks. This method has been widely used with different modifications. For instance, it was shown that KA, injected subcutaneously, causes similar behavioral and network alterations as an intraperitoneal injection (Sperk et al., 1983) and can be used as a model for TLE (Bertoglio et al., 2017). The significant advantage of systemic KA administration is its low labor-intensity, which allows the injection of numerous animals in a comparatively shorter time. Moreover, the absence of a surgical procedure eliminates side effects created by anesthesia, surgery invasiveness, and extra damage made by direct contact with brain tissue during the intracerebral injection. However, this model’s obvious disadvantages are (1) no control over the bioavailability of KA in the brain and (2) high mortality rates. As it was already mentioned before, Long–Evans and Wistar rat strains have higher mortality (Curia et al., 2008). In mice, C57 and CH3 strains display an increased mortality rate, while 129/SvJ and SvEms strains have a higher ratio of survival (McKhann et al., 2003). The summarized data for systemic KA administration in mice and rats is presented in Tables 2, 3. It is important to note that KA is quite expensive (e.g., as compared with pilocarpine) and that the purity of the molecule can vary from one stock to another (personal experience). There are also different forms of the drug, e.g., pure acid and its dehydrate, which can sometimes be an issue in terms of result variability. Periods of KA shortage were also experienced in the past.

**Table 2:** Post-KA chronic epilepsy in rats

Administration mode	SE	Latent period	SRS	Comorbidities	References
Systemic	Starts within 2 h Lasts for 5–9 h 22% mortality	12–36 d	8 per day (±5.4)	Cognitive impairment Aggressiveness	Stafstrom et al. (1992) Lado (2006) Williams et al. (2009) Drexel et al. (2012) Bertoglio et al. (2017)
ICV	Starts within 10–30 min Lasts for 2–6 h <10% mortality	7–14 d	5 per day (±3.5)	Cognitive impairment	Miyamoto et al. (1997) Jing et al. (2009) Gordon et al. (2014) Song et al. (2016) Gao et al. (2019)
Intra-HC	Starts within 5–20 min Lasts for 3–15 h <5% mortality	13–30 d	8 per day (±6.3)	Cognitive impairment Perseverative behavior Hyperexcitability	Cavalheiro et al. (1982) Bragin et al. (2005) Arkhipov et al. (2008) Rattka et al., 2013) Klee et al. (2017)
Intra-Am	Starts within 5–30 min Lasts for 4–6 h 10% mortality	11–24 d	12 per day (±6.2)	No data	Ben-Ari et al. (1979) Tanaka et al. (1992) Ueda et al. (2001) Takebayashi et al. (2007) Gurbanova et al. (2008)

Am, amygdala; HC, hippocampus; ICV, intraventricular; SE, status epilepticus; SRS, spontaneous recurrent seizures

**Table 3:** Post-KA chronic epilepsy in mice

Administration mode	SE	Latent period	SRS	Comorbidities	References
Systemic	Starts within 15–30 min Lasts for 2–6 h 27% mortality	2–3 d	Rarely observed Infrequent	Hyperactivity Delayed mortality	Hu et al. (1998) McKhann et al. (2003) Benkovic et al. (2004) Puttachary et al. (2015) Umpierre et al. (2016)
Intra-HC	Starts within 30 min Lasts for 3–12 h <5% mortality	2–14 d	1–2 per week Highly variable eSRS+++	Cognitive impairment Hyperexcitability Depression (lesioned ventral hippocampus)	Bouilleret et al. (1999) Riban et al. (2002) Gouder et al. (2003) Gröticke et al. (2008) Lee et al. (2012) Zeidler et al. (2018)
Intra-Am	Starts within 20 min Usually terminated at 30–40 min No mortality	3–12 d	3 per day (±2.5)	Anxiety	Araki et al. (2002) Shinoda et al. (2004) Mouri et al. (2008) Tanaka et al. (2010) Li et al. (2012) Liu et al., 2013)
Supra-HC	Lasts for 2–7 h <10% mortality	4–13 d	2.8 per day (±3.5)	No data	Bedner et al. (2015) Jefferys et al. (2016) Pitsch et al. (2019)
Intranasal	Starts within 15–30 min Lasts for 1–5 h ∼6.5% mortality	15–30 d	Reported	Increased locomotion	Chen et al. (2002) Duan et al. (2006) Zhang et al. (2008) Lu et al. (2008) Sabilallah et al. (2016)

Am, amygdala; eSRS, electrographic SRSs; HC, hippocampus; SE, status epilepticus; SRS, spontaneous recurrent seizures

#### SE

The sequence of events, followed by KA administration, includes the “initial insult,” e.g., SE, a latent seizure-free period, and the eventual development of chronic epilepsy. SE is characterized as a condition resulting either from the failure of the mechanisms responsible for seizure termination or the initiation of events, leading to abnormally, prolonged seizures (after time point *t* 1; El Houssaini et al., 2015). It is a condition, which can have long‐term consequences (after time point *t* 2), including neuronal death, neuronal injury, and alteration of neuronal networks, depending on the type and duration of the status (Trinka et al., 2015). In adult rats, a single dose of KA can trigger SE characterized by a catatonic posture followed by facial myoclonus (stage 1; according to Racine scale, Racine, 1972), masticatory automatisms, wet-dog shakes and head nodding (stage 2), rearing with facial automatisms and forelimb clonus (stages 3–4), and finally repeated rearing and falling (stage 5; Raedt et al., 2009). Following systemic KA administration, SE develops within two h and lasts for ∼5–9 h (if not stopped with an *ad hoc* injection of a drug), according to multiple experiments on rats (Stafstrom et al., 1992; Giorgi et al., 2005; Lado, 2006; Williams et al., 2009; White et al., 2010; Drexel et al., 2012; Bertoglio et al., 2017). Mortality in this model is relatively high (∼22%), which can be reduced with slight alterations of the protocol. Repeated injections of 2.5 mg/kg intraperitoneally (Hellier and Dudek, 2005) at 30-min intervals reduce mortality down to ∼5–6%, as compared with a single full-dose injection. Likewise, to increase survival rate, SE can be decreased in severity after a few hours by subcutaneous injection of 10 mg/kg diazepam or zolazepam (Gurbanova et al., 2008; Sharma et al., 2008). Many groups try to stop SE after 20 min. As mentioned above, control of SE duration requires instrumenting the animals for EEG recordings. We found a decreased mortality and accelerated recovery when timing SE with electrophysiological recordings (as opposed to timing SE duration with behavior). We are using the protocol to instrument animals with a telemetry device for supradural recordings at least two weeks before KA injection. Two to three days before KA injection, we start EEG recordings in the home cage, including following KA injection.

In mice, the clinical picture of systemically induced SE is similar to those seen in rats with slight differences and nuances. For instance, mice do not always fit in the Racine scale; “wet-dog shakes,” while being common for rats, are never observed in mice (Sharma et al., 2018). The behavioral manifestation of SE occurs within 15–30 min postinjection (Hu et al., 1998; Benkovic et al., 2004). The duration of SE does not exceed 6 h (McKhann et al., 2003; Puttachary et al., 2015). The mortality of systemic KA administration for mice is relatively significant (∼27%) and can be similarly reduced with repeated injections of 5 mg/kg intraperitoneally (Tse et al., 2014). Anticonvulsant injections are likewise efficient in reducing the severity of SE (Araki et al., 2002).

One important parameter to consider when inducing SE is the time of the day. Human studies show that SE occurs at specific times during the night and day cycle (Sánchez Fernández et al., 2019; Goldenholz et al., 2018). Since the molecular architecture of the hippocampus (and other brain regions) oscillates in a circadian manner, the sensitivity to SE may also depend on the hour of the day (Debski et al., 2020; Bernard, 2021). Performing injections spread over several hours in a given day may lead to variable outcomes, not only in terms of survival but also in terms of phenotype.

The KA-induced SE correlates with epileptogenesis. Although it is intuitively straightforward to propose that SE triggers epileptogenesis, it is still possible that the same cause triggers SE and epileptogenesis, i.e., SE does not cause epileptogenesis per se. There is some variability in terms of SE severity and recovery between individual rodents, which may be because of basal biological differences (Manouze et al., 2019), including anxiety levels, social hierarchy, etc. This comment is also valid for all other KA models described hereafter.

#### Latent period and SRSs

The latent period is the time between the initial brain insult (SE in the case of the KA rodent model of TLE), and the development of chronic epilepsy manifested as the first SRSs. The latent phase is considered one of the hallmarks of human epilepsy, and it can last for many years (French et al., 1993; Mathern et al., 1995). In some cases of epilepsy induced by TBI, stroke, or meningitis, the latent period can be as short as one week (Löscher et al., 2015). The variability of the latent period in humans constitutes a major puzzling question. It would be interesting to determine whether the phenotype depends on the duration of the latent period. Alternatively, since seizures are physiological or built-in activities in any normal brain (Jirsa et al., 2014), the problem can be rephrased as a probability one. The brain insult may trigger reorganizations that will evolve on slow and fast time scales to progressively increase the probability for spontaneous seizures. The path taken to SRSs may be traveled at different speeds, but it could be the same. This is difficult to model in rodents. As previously mentioned, several studies report that some animals never developed SRSs after SE. However, animals were never recorded 24/7 for months. It is possible that some animals develop SRSs after a very long-lasting latent period. Such models would be particularly useful to address the question of the variability of the latent period in humans.

In the rat model, the usual duration of the seizure-free period is ∼5–30 d (White et al., 2010; Chauvière et al., 2012; Drexel et al., 2012), although in some cases, it may take up to five months (Williams et al., 2006). Experimental animals do not exhibit any behavioral seizures during this phase, but electrophysiological recordings may show some epileptic activity, e.g., non-convulsive seizures, which will be described next. Whether a “true” latent period exists is debated, as the EEGs display evident anomalies in the few hours following SE, particularly interictal-like spikes. In fact, the latent period’s definition can be challenging as variability between recording methods, disagreement on the definition of convulsive seizures, and even the differences in electrode placement create certain obstacles. Some studies considered only behavioral seizures to be the hallmark of SRSs and the end of the latent period, while others claimed that the presence of electrographic seizures is the beginning of the chronic phase. Thus, the data on the latent period differ greatly. In our hands, pathologic activities, as manifested by spikes and bursts of spikes, appear soon after SE induction and never stop, even after the first spontaneous seizure (Chauvière et al., 2012).

The appearance of SRSs can be considered as the onset of the chronic phase of epilepsy. The average daily frequency of seizures is eight per day (±5.4; Stafstrom et al., 1992; Giorgi et al., 2005; Lado, 2006; Williams et al., 2009; White et al., 2010; Drexel et al., 2012; Bertoglio et al., 2017). SRSs developing following KA injection in rats at different ages last for ∼40 s. They resemble stage five limbic seizures induced by electric kindling: bilateral forelimb clonus, masticatory movements, rearing, and falling (Stafstrom et al., 1992). Subsequent studies, using continuous EEG/video recording, demonstrated similar results, with the seizure ratio varying from 4 to 21 per day (Lado, 2006; Williams et al., 2009). The F344 rat strain has been demonstrated to be sensitive to KA with a much lower dose (10.5 mg/kg) needed to induce SRSs (Rao et al., 2006). Prominent neurodegeneration in the hippocampus, extensive mossy fiber sprouting, and the consistent SRS ratio, with the same frequency over time, makes this strain interesting to obtain a stable and reproducible model.

In mice, the latent phase usually is significantly shorter than in rats and could be estimated as 2–3 d (Puttachary et al., 2015). Unlike the rat model, the development of SRSs is more difficult to obtain in mice following a systemic administration, and those detected are infrequent and highly variable (McKhann et al., 2003; Umpierre et al., 2016).

It is important to note that the high rate of SRSs reported in previous studies may be because animals are singly housed, which increases seizure frequency by a factor of 16, at least in the pilocarpine model (Bernard, 2019; Manouze et al., 2019). Single housing adds stress as an independent variable and a confounding factor to the epilepsy phenotype. We recommend maintaining social interaction (Bernard, 2019Manouze et al., 2019). This comment applies to all models.

Take home message #4: it would be particularly informative to perform a classification of pathologic events in each model, particularly spikes and seizures, using unsupervised analysis. As spikes and seizures may evolve in time, it is important to describe their dynamics during epileptogenesis and later. This comment is also valid for all other KA models described hereafter.

#### Histologic evaluation

Systemic administration of KA induces extensive bilateral neuronal damage throughout the brain. The first noticeable changes appear within 48 h posttreatment and are present primarily in CA1, CA3, and CA4 hippocampal subregions (Sperk et al., 1983; Haas et al., 2001; Sommer et al., 2001; Drexel et al., 2012). Subsequently, the entire hippocampus is affected in the rat brain (Kar et al., 1997; Suárez et al., 2012). The typical pattern of KA-induced damage includes necrosis of pyramidal cells, gliosis, and mossy fiber sprouting within the dentate gyrus’ inner molecular layer (Tauck and Nadler, 1985). Additionally, there have been multiple reports indicating damage of various extrahippocampal areas, such as the amygdala (Fritsch et al., 2009), subiculum, entorhinal cortex, thalamus, caudoputamen (Drexel et al., 2012), substantia nigra, hypothalamus (Covolan and Mello, 2000), olfactory bulb, anterior olfactory nucleus (Anthony Altar and Baudry, 1990), and other areas.

T2-weighted MRI images of Wistar and Sprague Dawley rat strains, systemically treated with KA, showed an interesting phenomenon, contradicting previous mortality findings. The extent of neuronal damage is higher in the Sprague Dawley strain, while in Wistar rats, the relative volume of the hippocampus is not different from the control animals (Inostroza et al., 2011). Confirming the MRI data, postmortem NeuN-staining revealed a significant pyramidal loss in CA1 and CA3 areas of the hippocampus in Sprague Dawley rats but not in Wistar (Table 1).

In mice, the predominantly affected areas are CA3 and CA1 (Hu et al., 1998; McKhann et al., 2003; Kasugai et al., 2007; Mouri et al., 2008). Similarly to rats, damage induced by systemic KA administration includes pyramidal cell loss, mossy fiber sprouting, and reactive gliosis (McKhann et al., 2003; Benkovic et al., 2004). There is also evidence of extratemporal damage, particularly in the cortical areas, lateral amygdala, and dorsal thalamus (Hu et al., 1998; McKhann et al., 2003; Benkovic et al., 2004; Umpierre et al., 2016).

Take home message #5: as noted above, a better understanding of all morphologic changes in different brain regions is needed to obtain a global picture of the model. This comment is also valid for all other KA models described hereafter.

### Intraventricular injection

Nadler et al. (1978) have demonstrated that the intracerebroventricular (i.c.v.) injection of 0.5 nmol KA into the rat brain leads to CA3 neurodegeneration within 1–3 d, whereas higher doses (0.8 mg and more) cause damage to both CA1 and CA2. Similar findings were later observed by Lancaster and Wheal, 1982; who confirmed the same neuronal damage pattern and helped establish the i.c.v. paradigm, which is still used (Luo et al., 2013; Song et al., 2016). The data of i.c.v. KA administration in rats is presented in Table 2.

#### SE

The induction of SE using the i.c.v. route is consistent with other methods. It starts within 10–30 min postinjection and manifests as bradypnea, circling behavior, and wet-dog shakes and subsequently progresses into continuous motor seizures (Miyamoto et al., 1997). The duration of SE varies between 2 and 6 h, which can be stopped by a diazepam injection to reduce the mortality rate (Miyamoto et al., 1997; Jing et al., 2009; Song et al., 2016). Typically, the mortality rate does not exceed 10% and is considered relatively low compared with the systemic route of administration (Miyamoto et al., 1997; Gao et al., 2019).

#### Latent period and SRSs

There are multiple reports regarding SRS development following i.c.v. KA administration. Having performed a 24/7 continuous video/EEG recording, few authors reported similar findings, with the latent period duration varying between one and two weeks and SRSs appearing consistently, several times per day (Song et al., 2016; Gao et al., 2019).

#### Histologic evaluation

Morphologic damage induced by an i.c.v. KA administration might seem paradoxical; although the toxin is injected in the ventricular system and thus potentially distributes throughout the brain, the actual lesion appears to be restricted to CA3/C4 hippocampal subfields ipsilaterally to the injection site (Nadler et al., 1978; Miyamoto et al., 1997). The contralateral hippocampus and the extrahippocampal areas are relatively spared, and in some cases, almost entirely intact (Song et al., 2016). Mossy fiber sprouting is also present in most cases (Jing et al., 2009; Song et al., 2016; Gao et al., 2019). The pathology is mostly unilateral, with occasional signs of damage on the contralateral side (Lancaster and Wheal, 1982; Miyamoto et al., 1997). The model is closer to what is found in patients with MTLE, as the epileptogenic network is usually limited to one hemisphere (Table 1).

### Intrahippocampal and intra-amygdaloid injections

The first experiment with intra-amygdaloid KA administration in 1978 demonstrated characteristic behavioral, electrophysiological, and histologic hallmarks in baboons (Ben-Ari and Lagowska, 1978; Ben-Ari et al., 1979). Other studies used intrahippocampal KA injections in rats to observe the same outcome (Fonnum and Walaas, 1978). Both methods of intracerebral administration are still widely used. The principal advantage is a direct delivery into the brain tissue, bypassing the blood-brain barrier. The idea is to produce focal damage. The mortality rate appears to be lower than systemic administration (Sharma et al., 2007). Moreover, the standardized protocol for the intrahippocampal KA administration, combined with the electrode implantation, which has been proposed recently (Bielefeld et al., 2017), enables a good reproducibility and allows the combination of both electrophysiological and behavioral approaches. A brief summary of both methods is presented in Tables 2, 3.

#### SE

SE, following an intracerebral KA injection, develops rapidly. Experiments showed that in rats, which underwent intrahippocampal KA administration in the dose of 0.4–2.0 μg, SE emerges within 5–30 min after the injection (Bragin et al., 1999; Raedt et al., 2009). Similar effects were observed after the intra-amygdaloid injection of 0.75 μg of KA (Gurbanova et al., 2008). SE, elicited by intrahippocampal administration appears to be longer than systemic administration and can last for >17 h (Rattka et al., 2013). Intra-amygdaloid KA administration in rats is efficient in triggering SE lasting 4–6 h on average (Ben-Ari et al., 1979; Tanaka et al., 1992). Generally, intracerebral administration’s lethality does not exceed 10% (Rattka et al., 2013).

Mice demonstrate the same pattern of SE starting within 30 min after intrahippocampal (Lee et al., 2012) or within 20 min after intra-amygdaloid (Mouri et al., 2008) administration. The duration is approximately the same as for rats and can last for up to 12 h (Gröticke et al., 2008). However, there is a common approach to termination seizures via an anticonvulsant injection after 40 min of seizure activity, thus reducing mortality rate to a minimum (Shinoda et al., 2004). FVB/N mouse strain exhibits prolonged seizure activity as compared with C57BL/6 strain after intra-amygdaloid KA administration, although the overall SE pattern is similar in both strains (Kasugai et al., 2007). The mortality rate is relatively low compared with the systemic mode of drug administration (Bouilleret et al., 1999; Mouri et al., 2008).

#### Latent period and SRSs

One of the first successful experiments on intrahippocampal KA administration was performed by Cavalheiro et al. (1982), when he and his colleagues conducted a series of tests in rats, using several different doses of KA in the range of 0.1–3.0 μg, thus creating three distinct cohorts: animals which were injected with either 0.1–0.4 or 0.8–2.0 μg experienced SE, but only the latter group developed SRSs ∼5–21 d after treatment. The rats injected with 3.0 μg of KA died because of severe SE. The SRSs resembled those happening during the acute phase: lasting for 40–60 s, involving salivation, masticatory movements, bilateral forelimb clonus, rearing, and falling. The chronic period continued for 22–46 d, after which behavioral seizures ceased, and animals entered a postseizure period. Generally, the occurrence of SRSs after the intrahippocampal administration of KA is consistent in rats and resembles the pattern of systemic injection (Rattka et al., 2013).

Intra-amygdaloid administration is efficient for inducing chronic epilepsy in rats. The average amount of intrahippocampal KA-induced SRSs is 12 per day (Gurbanova et al., 2008). Latency to the first SRS is reported to be one to four weeks (Cavalheiro et al., 1982; Klee et al., 2017) in both intrahippocampal and intra-amygdaloid administration.

The first experiment on intrahippocampal KA injection in mice was performed in 1999, and SRSs were observed with the frequency of one to two seizures per week (Bouilleret et al., 1999). Unlike rats, the EEG results in mice are highly variable, with some authors reporting the frequency of electrographic SRSs to reach dozens per hour (Riban et al., 2002; Gouder et al., 2003), which could be because of social isolation-induced stress (Bernard, 2019; Manouze et al., 2019). According to a recent study, this phenomenon of frequent electrographic seizures is specific to mice but not to rats (Klee et al., 2017).

It is important to highlight the study of Sheybani and colleagues (Sheybani et al., 2018). To the best of our knowledge, with the study performed in the pilocarpine model (Toyoda et al., 2015), this is the only other study in which multisite recordings have been performed to assess an experimental model of TLE at the large-scale network level. The study clearly shows that epileptiform activity progressively expands beyond and independently from the seizure focus. This result is particularly important as it clearly shows that time-dependent alterations in neuronal circuits need to be considered when studying epilepsy in chronic experimental models, including during epileptogenesis (Słowiński et al., 2019).

Following the intra-amygdaloid KA administration, SRSs developing in mice have a frequency of one to five per day (Mouri et al., 2008; Tanaka et al., 2010; Li et al., 2012; Liu et al., 2013). The latent period does not exceed two weeks for both types of intracerebral administration (Mouri et al., 2008; Lee et al., 2012).

An interesting phenomenon was noticed following an intrahippocampal KA administration in mice. The dentate gyrus is the major zone of adult neurogenesis (Hochgerner et al., 2018). Normally, systemic administration of KA induces bilateral neurogenesis in the hippocampus, which, combined with the data collected from human patients, led to an assumption that increased neurogenesis might contribute to epileptogenesis in TLE (Parent, 2007). However, findings in the intrahippocampal KA mouse model of TLE suggest the opposite. Arabadzisz et al. (2005) demonstrated that KA, unilaterally injected into the hippocampus, causes a massive reduction of the neurogenesis on the injection site and a compensatory neurogenic activity in the contralateral dentate gyrus. It could be speculated that, according to these data, neurogenesis does not contribute to the genesis of the epileptogenic zone and that, perhaps, it gets disrupted by changes induced in the hippocampus, e.g., granule cells dispersion. This topic is interesting to pursue. A useful review on this topic was published in 2012, and it partly covers the KA model (Parent and Kron, 2012).

#### Histologic evaluation

**Figure 3.**
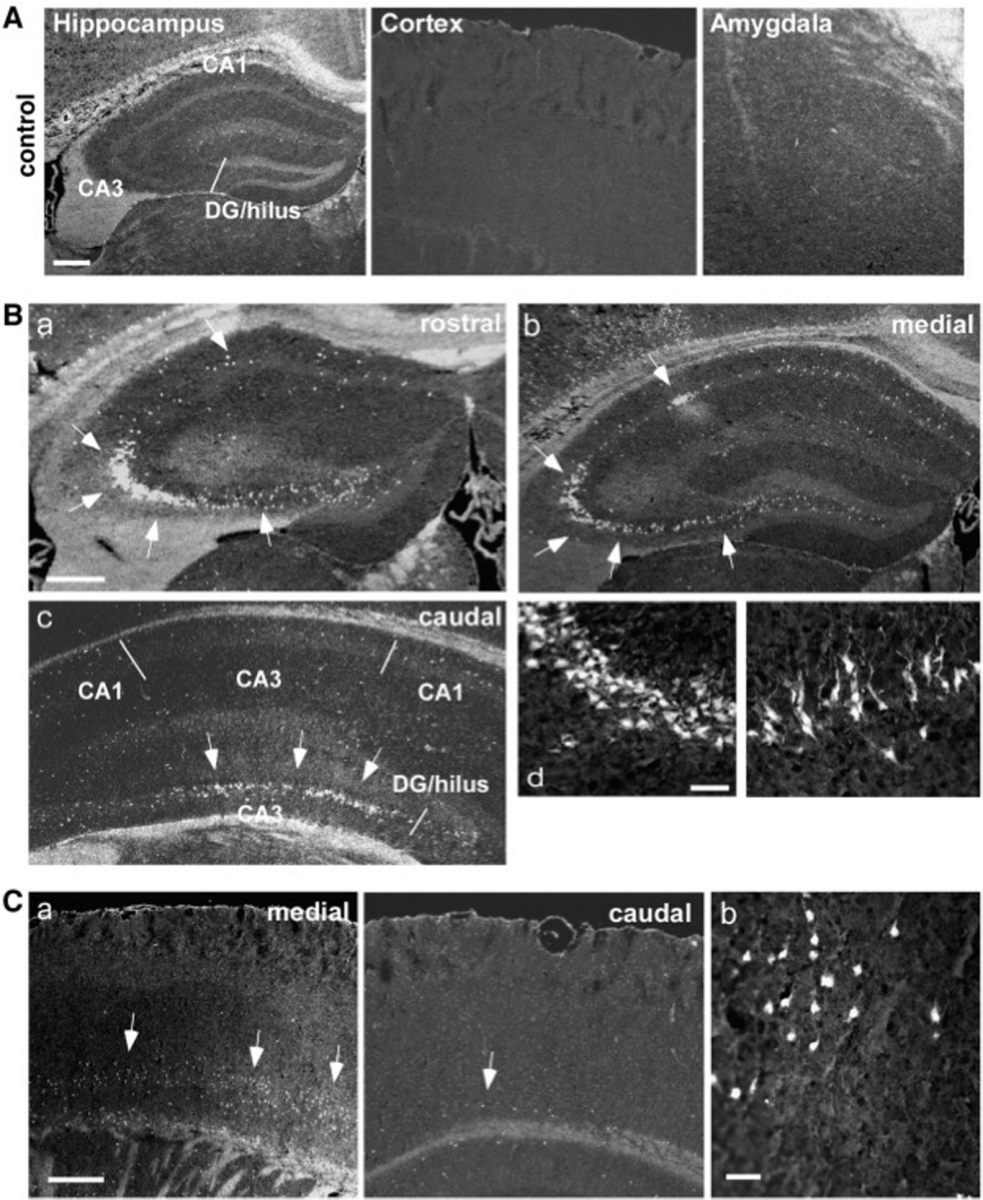
Neuropathological alterations in hippocampal and cortical areas followed by intra-amygdaloid KA administration, FluoroJade B (FjB) staining. ***A***, Hippocampus, cortex and amygdala of a control mouse. Absence of FjB-positive cells. ***B***, Images of ipsilateral hippocampus of a KA-treated mouse at anterior, median and posterior levels. FjB-positive cells are indicated by arrows. ***C***, Representative images of ipsilateral temporal cortex of a KA-treated mouse. (Mouri et al., 2008).

Histologic studies reveal similarities between the two intracerebral KA administration routes, which are fairly similar in rats and mice (Table 1). The intrahippocampal injection creates a focal lesion in the hippocampus, damaging predominantly the ipsilateral CA3 subfield and, to some extent, CA4 and CA1, regardless of the site of injection (French et al., 1982; Bouilleret et al., 1999; Raedt et al., 2009; Twele et al., 2015). Interestingly, intra-amygdaloid administration induces massive neuron destruction in the same hippocampal regions, along with a lesion in the amygdala (Tanaka et al., 1992; Araki et al., 2002). Some authors reported extensive damage on the contralateral side (Ben-Ari et al., 1980; Araki et al., 2002; Mouri et al., 2008) and the resistance of the CA2 hippocampal subfield to the KA-induced toxicity (Shinoda et al., 2004). Mossy fiber sprouting was also observed in both rats and mice (Gurbanova et al., 2008; Mouri et al., 2008; Raedt et al., 2009; Zeidler et al., 2018). Granule cell dispersion in the dentate gyrus, however, is only reported with the intrahippocampal administration (Rattka et al., 2013; Bielefeld et al., 2017). Additionally, there is multiple evidence of damage in extratemporal regions induced by intra-amygdaloid injection, such as thalamus (Ben-Ari et al., 1980), entorhinal, and perirhinal cortices (Mouri et al., 2008; Tanaka et al., 2010). There is a significant strain difference in mice. The C57BL/6 mouse strain is resistant to cellular damage induced by KA (Schauwecker and Steward, 1997; Kasugai et al., 2007). In contrast, the FVB/N strain shows increased sensitivity and extensive neurodegeneration in the CA1 and CA3 areas of the hippocampus following intra-amygdaloid KA administration (Kasugai et al., 2007). A representative image of the FluoroJade B (FjB) staining of murine hippocampus and cortex after intra-amygdaloid KA injection is presented in Figure 3. It has to be stated that the figure does not represent all the possible variations in histologic findings but only a particular case. The variability of KA-induced damage is high, and it should be assessed with a stereological method, if possible. Since it is difficult to summarize the data pool regarding histologic studies, we chose only one image.

### Suprahippocampal

One of the variations of the intrahippocampal administration route is the so-called suprahippocampal injection, where KA is injected in the cortical area just above the hippocampus. This technique was introduced by Bedner et al. (2015), who performed an intracortical injection to reproduce the features of the intrahippocampal administration and avoid damaging the CA1 subfield of the hippocampus. All animals experienced SE, triggering epileptogenesis. Subsequently, other researchers tested this method several times, proving to be efficient in mice (Table 3).

#### SE

Starting soon after recovery from anesthesia, SE induced by the intracortical KA application lasts for up to 12 h and consists of forelimb clonus and repetitive rearing and falling (Jefferys et al., 2016). The reported mortality is relatively low and ranges from 0% (Pitsch et al., 2019) to 9.7%. As mentioned by the authors, death occurred mostly because of surgical complications and not seizures (Bedner et al., 2015).

#### Latent period and SRSs

SRSs in this model appear after a brief latent period of 4–13 d and are consistent and frequent. Bedner et al. (2015) reported a frequency of 7.5 ± 6.2 seizures per day seven to nine months post-SE and progressive nature of chronic epilepsy development, consistent with previous data on KA-induced seizure progression (Williams et al., 2009). Another study demonstrated success inducing SRSs in 100% of treated animals with the seizure frequency of 1–1.5 per day (Pitsch et al., 2019).

#### Histologic evaluation

The lesion created by suprahippocampal injection of KA is unilateral and predominantly situated in the CA1/CA3 hippocampal subfields. Three months posttreatment, there is a complete neuronal loss in CA1, significant damage in CA3, and massive dentate granule cell dispersion (Bedner et al., 2015; Jefferys et al., 2016; Pitsch et al., 2019). Mossy fiber sprouting is not characteristic of this model (Table 1).

It would be particularly interesting to determine whether the epileptogenic network develops as in the intrahippocampal model (Sheybani et al., 2018).

### Intranasal injection

The idea of delivering KA via nasal epithelium absorption first emerged as an attempt to trigger SE in the most common strain of transgenic laboratory mice, C57BL/6, known for its resistance KA-induced neuronal death (Schauwecker and Steward, 1997). The first experiments showed that KA, dissolved in water and delivered intranasally in the dose of 40–60 mg/kg, causes focal seizures, which then generalize into SE, in 100% of treated C57BL/6 mice (Chen et al., 2002). Behavioral and anatomic findings were consistent with the previous results, obtained via standard drug delivery protocols. Likewise, another study demonstrated that a dose of 30 mg/kg KA intranasally is associated with low mortality and >90% success in developing SE (Sabilallah et al., 2016). Although the mechanism is not fully understood, it is assumed that the drug is absorbed by olfactory epithelium and reaches the hippocampus (as well as other brain areas) via olfactory pathways (Frey et al., 1997; Fig. 1*C*), as the olfactory bulb has widespread connections with different regions of the brain (Chen et al., 2002). Although this method has not been studied thoroughly and so far has not found widespread success in the neuroscience community, it has its advantages, such as reproducibility, low labor intensity and low mortality (Table 3).

#### SE

The induction of SE appears to be consistent in all existing studies; it starts within 15–30 min after administration (Chen et al., 2002; Duan et al., 2006) with the first symptoms being immobility and staring, followed by generalized tonic-clonic seizures, lasting for 1–5 h (Chen et al., 2002). The mortality rate is relatively low and can be compared with intrahippocampal routes, ranging from 0% to 12% (Chen et al., 2002; Duan et al., 2006; Lu et al., 2008; Zhang et al., 2008; Sabilallah et al., 2016). Generally, there are no specific SE features associated with the intranasal administration route compared with the conventional methods.

#### Latent period and SRSs

Seizure progression was reported by at least one research group. Sabilallah et al. (2016) reported latency of 15–30 d before electrographic SRSs are observed in most animals in the form of spontaneous spike activity and occasional seizures. However, the study lacked continuous 24/7 video/EEG recording, so the actual outcome might slightly differ. Other authors reported increased locomotor activity (Chen et al., 2002; Lu et al., 2008; Zhang et al., 2008), perhaps in relationship with the development of chronic epilepsy.

#### Histologic evaluation

Morphologic damage induced by intranasal KA administration has been studied. The brain remains mostly intact, except for the hippocampus and the olfactory bulbs (Chen et al., 2002; Zhang et al., 2008). The CA3 area of the hippocampus shows the most prominent damage (Duan et al., 2006; Zhang et al., 2008). Mossy fiber sprouting and granule cell dispersion have not been reported. There is evidence of massive astrogliosis and microglial response in the hippocampus (Chen et al., 2002; Duan et al., 2006; Lu et al., 2008; Zhang et al., 2008; Sabilallah et al., 2016). Overall, there is evidence for characteristic pathologic features of “classic” KA-induced epilepsy. However, the damage seems to be localized and restricted to the CA3 area of the hippocampus (Table 1).

## Electrophysiology

SE elicited by KA administration is characterized by typically isolated spikes, polyspikes, spike and wave complexes (Stafstrom et al., 1992; Riban et al., 2002; Jagirdar et al., 2015). With the advantage of simultaneous EEG and video recording, it is possible to distinguish electrographic SE (ESE) from the convulsive SE (CSE), since numerous studies reported ESE to last much longer than CSE (Fritsch et al., 2010; Drexel et al., 2012; Sabilallah et al., 2016; Umpierre et al., 2016). Considering 20 min of ESE rather CSE results in better survival and more rapid recovery. However, this requires instrumenting the animals. It is important to remember that any invasive procedure (like implanting electrodes/transmitters under anesthesia) may change an unknown number of biological parameters in each experimental animal. In other words, we do not know whether instrumenting animals produces a different model/phenotype as compared with non-instrumented animals.

In our hands, after a drug injection to stop SE, we observe the occurrence of spikes, which frequency decreases during the first hours after SE, while the EEG slowly returns to the pre-SE level (between spikes). Then, the spikes start to organize themselves in bursts. During this phase, we see epileptiform discharges, which look like very short seizures (2 s long). A few days after SE, SRSs start to occur with a typical >10-s duration. In the pilocarpine model, two types of interictal spikes can be distinguished during the latent period: type 1 is a spike followed by a long-lasting wave, and type 2 is a spike without a wave (Chauvière et al., 2012). The authors suggest that type 1 spikes correspond to the activity of both excitatory and inhibitory neurons, while type 2 spikes reflect the activity of a small pool of excitatory cells. While the number, amplitude, and duration of type 1 spikes decreases while type 2 spikes become more frequent before the first spontaneous seizure. A similar study should be performed in the various KA models.

Spikes can evolve into hippocampal paroxysmal discharges (HPDs; Riban et al., 2002; Jagirdar et al., 2015), which are essentially short periods of epileptiform activity in the absence of any behavioral symptoms (Pallud et al., 2011). Latency for electrographic seizures is shorter than for convulsive seizures (Lado, 2006; Williams et al., 2009). During this time, occasional interictal spikes and HPDs can be observed (Riban et al., 2002; White et al., 2010).

The detection of electrographic SRSs precedes motor symptoms observation and does not always correlate to it (Bragin et al., 2005; Li et al., 2012; Tse et al., 2014).

**Figure 4.**
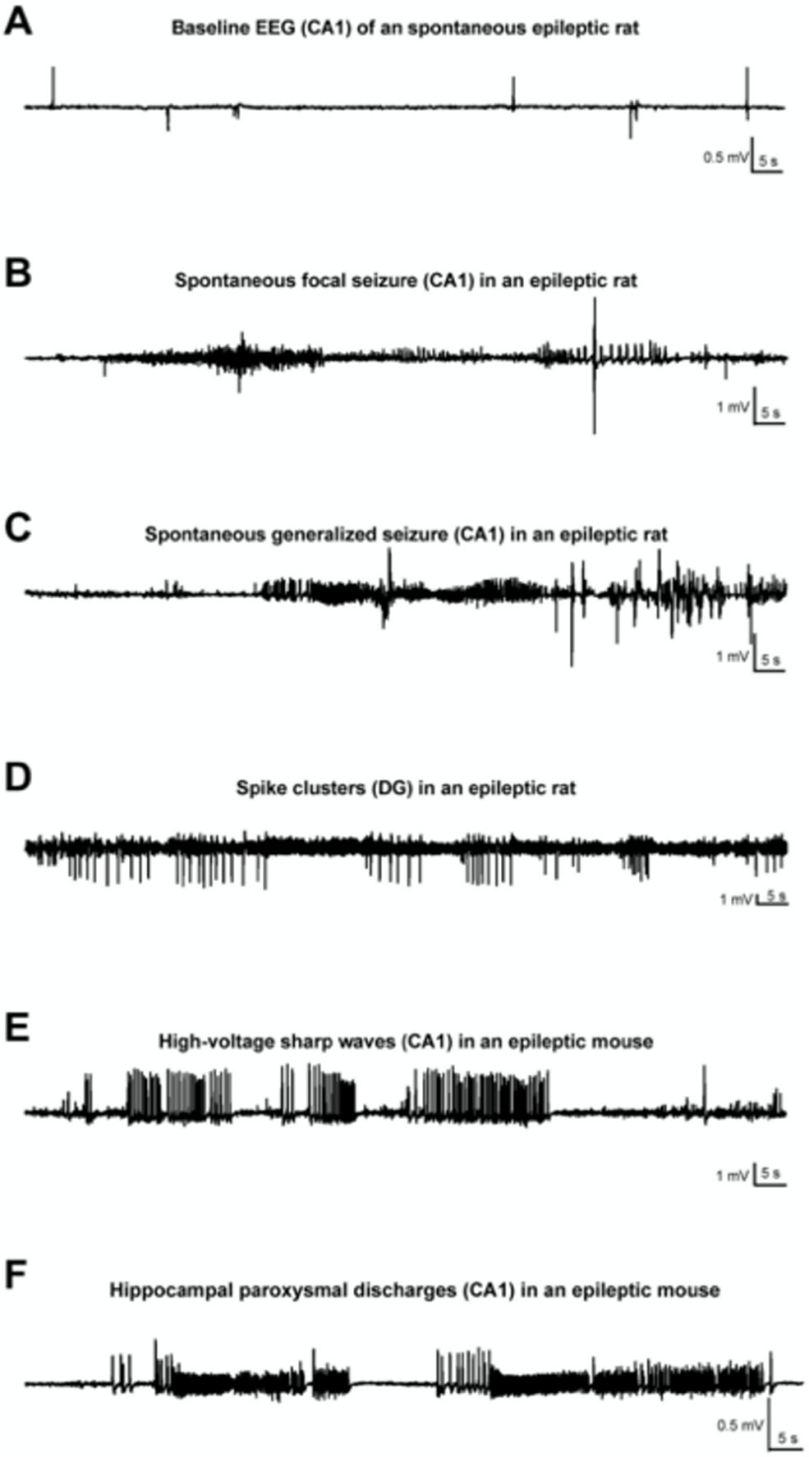
Various patterns of EEG-activity during KA-induced chronic epilepsy. ***A***, Baseline recording from CA1 of an epileptic rat. Note the occurrence of interictal spikes. ***B***, Recording of a spontaneous focal seizure in CA1. ***C***. A secondary generalized convulsive seizure in an epileptic rat. ***D***, Spike clusters originating from the dentate gyrus 7 weeks post-SE. ***E***, High-voltage sharp waves in the epileptic focus (CA1) of a mouse, several weeks post-SE. ***F***, Hippocampal paroxysmal discharges (HPDs) in CA1 of an epileptic mouse. (Klee et al., 2017).

Various electrographic patterns were reported in KA studies. For example, Riban et al. (2002) classified seizures emerging during the chronic period as low-voltage spikes (600–900 μV, 100–150 ms), which persisted for two weeks and were never observed again, bursts of high-frequency, low-voltage spikes (300–500 μV, 18–26 Hz), appearing just for 1–2 d, high-voltage sharp waves (1500–4500 μV, 150–200 ms), persisting until termination of the experiment, and HPDs, which, being the hallmark of the latent period, were still present during the chronic phase. Other authors (Bragin et al., 2005; Lévesque et al., 2012) reported two distinct seizure onset patterns for SRSs: HYP and LVF onset patterns, which are also found in patients with epilepsy (Velasco et al., 2000; Ogren et al., 2009). HYP seizures, which represent 50% to 70% of SRSs, are essentially multiple periodic spikes with a frequency of ∼2 Hz, restricted to a small portion in the hippocampus. LVF onset pattern consists of a single spike followed by high-frequency activity (>25 Hz) originating from hippocampal or extrahippocampal networks. The typical electrographic recordings of SRSs are presented in Figure 4. A taxonomy of 16 types of seizures has also been proposed and validated in patients (Jirsa et al., 2014; Saggio et al., 2020) and in the tetanus toxin model of epilepsy (Crisp et al., 2020), in which individual animals switch between different types of seizures during epilepsy. Future work is needed in the KA models to determine the type of seizures they express and how they evolve. This requires direct current recordings, as opposed to the most commonly used alternative current recordings.

**Figure 5.**
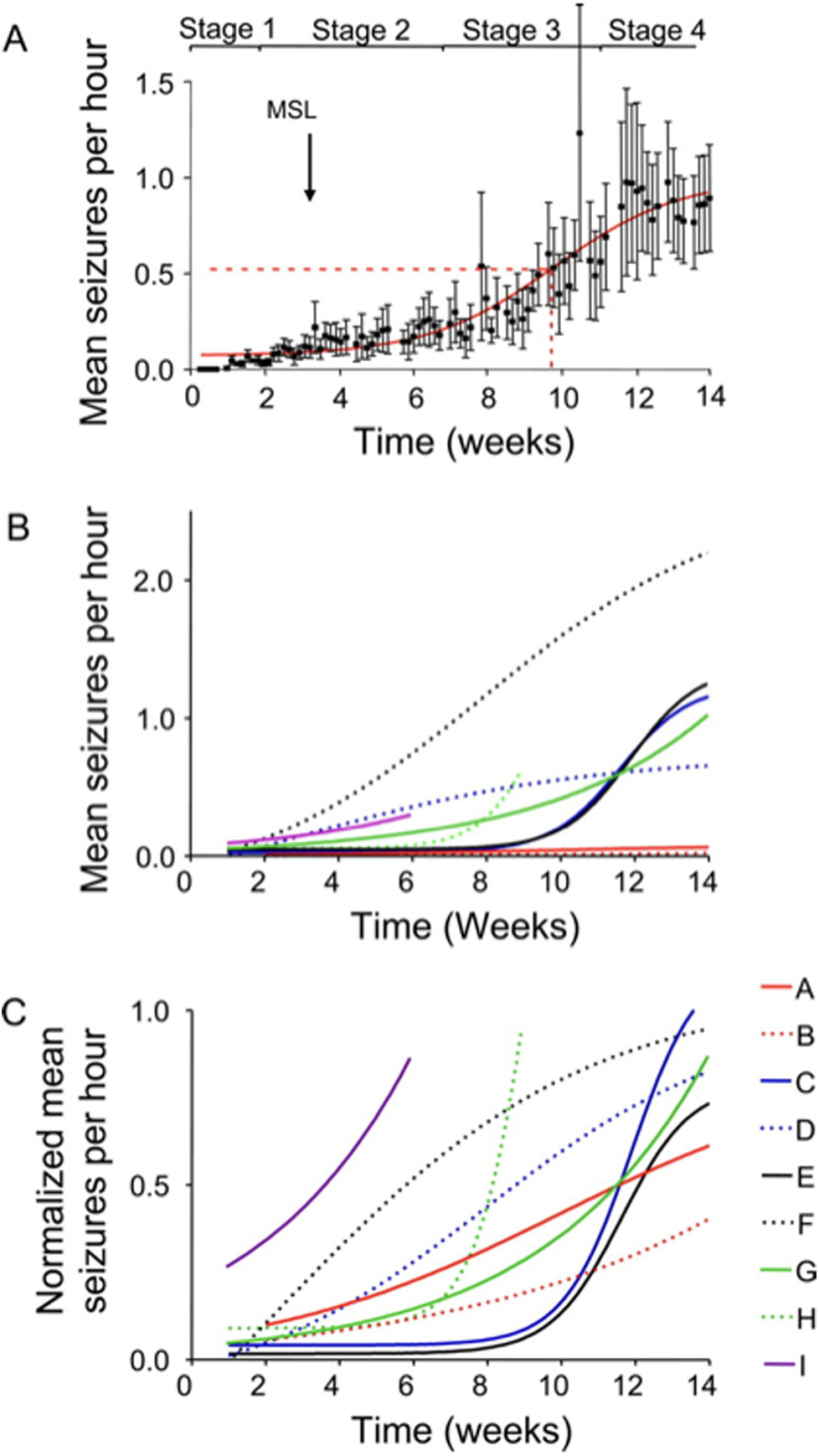
Seizure progression over time. ***A***, Increase in seizure frequency over 14 weeks post-injection. The latent period is reflected in stage 1, stage 2 represents the “slow growth phase”, stage 3 is characterised by an exponential growth until reaching the steady stage 4. ***B***, Actual seizure frequency plotted on the graph. Data obtained from the 9 animals. ***C***, Normalised seizure frequency, same cohort. (Williams et al., 2009).

The arguments developed previously clearly show complex dynamical phenomena that occur during the latent period. As soon as SRSs start to occur, it is essential to remember that a steady state is never reached. Seizures tend to progress over time (Table 2). Williams et al. (2009) proposed that the evolution of seizures consists of four stages, where stage 1 represents a latent period, stage 2 is characterized by a slow increase in seizure frequency, e.g., “slow growth phase,” stage 3 is marked as the “exponential growth phase,” and stage 4 is a final steady-state plateau phase, which, however, was not observed in all the animals (Fig. 5). This result agrees with other findings, most of which described a progressive increase of SRSs during the chronic period of KA-induced epilepsy (Gorter et al., 2001; White et al., 2010; Rattka et al., 2013; Van Nieuwenhuyse et al., 2015). Thus, the evolution of seizures can perhaps be best represented by a sigmoid Boltzmann function, showing the exponential growth phase. However, recent works depict a more complex picture as circadian and multidien rhythms need to be considered (Bernard, 2021).

Human studies clearly show that seizure activity follows various rhythms: circadian, ultradian, multidien or multiday, seasonal, etc. (Quigg, 2000; Manfredini et al., 2004; Spencer et al., 2016; Baud et al., 2018; Karoly et al., 2018). The circadian rhythmicity has been recognized for millennia in humans, but multidien rhythms have been identified recently in patients (Baud et al., 2018). The authors found that interictal activity, in addition to its circadian rhythmicity, has a slow envelope of several days period, which is patient specific. Interestingly, seizures tend to occur during the rising phase of this low rhythm. Besides, clusters of seizures are also found during the rising phase. Similar features have been found in the KA model with a robust periodicity of 5–7 d, each animal following its own cycle (Baud et al., 2019). Although rendering data analysis and interpretation more complex, it is important to take circadian and multidien cycles into account, particularly when investigating the molecular mechanisms underpinning seizure genesis (Debski et al., 2020). The fact that the molecular architecture of the hippocampus oscillates in a circadian manner (Debski et al., 2020), provides an entry point to understand the circadian rhythmicity of seizures (Bernard, 2021). It is important to stress again that KA’s sensitivity (e.g., the threshold to induce SE) may change during the night and day cycle (Debski et al., 2020).

Finally, as mentioned above, the way animals are housed appears as a key determinant of SRS frequency. When animals are singly housed, they are highly stressed, and their seizure frequency is 16 times greater than in animals maintaining social interaction (Bernard, 2019; Manouze et al., 2019). Solutions have been developed to maintain social interaction when animals are instrumented (Bernard, 2019; Manouze et al., 2019).

Take home message #7. Studying seizure frequency requires several weeks of 24/7 continuous recordings to account for circadian and multidien rhythms. Large discrepancies found in animals recorded during one week may stem from the fact that these animals were going through a high or low SRS frequency part of their multidien cycle.

## Age, Sex, and Strain Specificity

The outcome of KA administration depends not only on the used species and strains but also on the sex and age. This diversity is an advantage as it may allow us to reproduce the diversity of phenotypes found in TLE patients. For instance, C57BL/6 mice, the most widely used transgenic strain, are resistant to KA-induced damage, which can be, however, compromised by intranasal drug administration. Other strains with known resistance are BALB/c (Schauwecker and Steward, 1997), C3HeB/FeJ, 129/SvEms, 129/SvJ (McKhann et al., 2003), ICR, and FVB/N (McLin and Steward, 2006). It has also been proposed to differentiate between behavioral resistance (129/SvEms), lack of anatomic alterations (C57, C3H), and combined resistance (129/SvJ; McKhann et al., 2003).

In contrast, other mouse strains have increased sensitivity to KA, such as C57BL/10, DBA/2J, and F1 C57BL/6*CBA/J (McLin and Steward, 2006). Also, DBA/2J and FVB/N mouse strains exhibit higher seizure-induced mortality as compared with C57BL/6J (Ferraro et al., 1995; Royle et al., 1999). Overall, the difference between rodent strains is crucial and should always be taken into account. Investigating different outcomes in different strains could also help to understand the model better. In fact, not all researchers agree that KA, injected into a mouse brain, leads to chronic epilepsy development. Considering failures in inducing seizures followed systemic KA administration, inconsistent histologic data, and great variability between strains and even within one strain, it is necessary to point out that any result should be analyzed with care. However, current advances in science allow us to investigate the issue in more detail. Perhaps, over the next few years, there will be an agreement on whether mice indeed develop KA-induced epilepsy and how to interpret any related strain differences.

It is important to note that we have found a large variability between animal vendors in Europe for a given strain because the genetic background, the housing conditions, etc. may differ. For example, we experienced a major issue with several batches of animals from a given vendor. Our protocol was not working anymore. After probing, the vendor told us that they had changed the feeding conditions to accelerate weight gain. Since some injections are per kg, we were injecting a “much younger” animal. Besides, whatever they added to the food may have changed the biology of animals. This is also a source of variability and discrepancy between laboratories.

Another important factor one should consider when working with a rodent model of TLE induced by KA administration is the animals’ age. The first indications of different seizure susceptibility in younger and older rats appeared in the late 1980s when it was demonstrated that an intraperitoneal injection of KA in P12 rat pups causes more severe SE as compared with P27 adult rats (Holmes and Thompson, 1988). Subsequently, other authors reported similar results: shorter latency before developing SE in younger (P5–P10) rats compared with P20–P60 (Stafstrom et al., 1992), and higher mortality in P15 rats as compared with P53 after an intraperitoneal KA injection (Mikati et al., 2003).

Aged rodents are more prone to seizure activity than adults (Wozniak et al., 1991; McCord et al., 2008), and that this tendency does not depend on a strain (McCord et al., 2008). Aged rats have a shorter latency to SE onset and exacerbation of preseizure behavioral manifestations than middle-aged animals (Darbin et al., 2004). The reasons behind this are debated and include multiple theories including age-related changes in synaptic connectivity (Rapp et al., 1999), electrotonic coupling (Barnes et al., 1987), number and type of neurons (Stanley and Shetty, 2004) and diminished density of glutamate receptors in the aged brain (Wenk and Barnes, 2000; Lerma et al., 2001). Overall, these data seems to describe a parabolic pattern of KA-induced seizure susceptibility, where rodents of young and old age are more vulnerable than middle-aged animals. This correlates well with findings in human epilepsy, where age-specific incidence peaks in childhood and then, after a decline in middle age, raises again at the age of 60 years and older (Kotsopoulos et al., 2002).

An important point regarding age differences was already mentioned before and includes multiple models of TLE in immature rodents (Holtzman et al., 1981; Jensen et al., 1991; Dubé et al., 2010; Rakhade et al., 2011). Given the high prevalence of epilepsy during development and the fact that a developing brain is not a small adult brain, there is a dire need for specific developmental models of epilepsy, as information gained in adults does not translate automatically to the developing brain. Developmental epilepsy is a field in itself, and we refer the reader to the relevant literature, as we mostly focused here on adult TLE.

It is remarkable to notice that the vast majority of epilepsy studies have been and are being performed in male rodents. We do not know whether the wealth of data obtained in males applies to females. There is a dire need for research on female subjects of various strains. The currently available data demonstrate some clear differences between male and female rodents regarding the phenotype, epileptogenic processes in the brain, and anatomic alterations. Limiting research to male animals creates a possible misunderstanding of the model and epilepsy in general. This key issue remains to be addressed. Differences are expected, if only when considering structural arguments. The rodent hippocampus, particularly the CA3 area, contains various receptors for gonadal steroids. For instance, the concentration of androgen receptors in stratum lucidum of the CA3 subfield appears to be higher than in any other hippocampal region (Tabori et al., 2005), and mossy fibers express estrogen receptor (ER)α and ERβ (Torres-Reveron et al., 2009). Several studies have shown that mossy fiber pathway stimulation evokes different levels of brain-derived neurotrophic factor (BDNF) protein expression response in males, females in various stages of estrous cycle and ovariectomized female rats (Scharfman et al., 2003; Scharfman et al., 2007; Skucas et al., 2013). Only females in proestrous and estrous stages of menstrual cycles exhibit epileptiform activity after a 10-s 1-Hz train of paired pulses and exhibit a strong BDNF expression (Scharfman et al., 2003, 2007).

Furthermore, in C57BL/6 mice, KA injected at 20 or 30 mg/kg causes SE in ∼100% of aged female mice compared with the young females and males of both groups and the highest level of BDNF expression (Zhang et al., 2008). Altogether, these data provide a valuable insight into the influence of sex on KA administration and susceptibility to seizures, showing that the estrous cycle should be considered while modeling epilepsy in female mice. However, a tremendous amount of research effort is required to study epilepsy in experimental female TLE models.

## Concluding Remarks

In this review, we have described the different versions of the KA model. This model has been extensively used for decades and is proven to be a reliable tool to mimic numerous behavioral, electrophysiological, and anatomic features of epilepsy. In our opinion, whether or not the KA models are mimicking human TLE is not a relevant question if only because a rodent brain is not a human brain. Important are the questions addressed in the rodent models and the hypotheses being tested. We argue that all models are interesting as long as they are characterized by spontaneous seizures.

Diversity is a hallmark of human epilepsy, even for some specific types such as TLE. Interestingly, different KA models are also very diverse. The results vary as a function of how KA is administered, species, strain, sex, and age. We argue that this is a strength as the different models may cover some of the patients’ diversity.

Because diversity is the hallmark of human epilepsy, clinicians consider patients as individuals. We argue that a similar approach should be used with rodents. We have started to consider each rat or mouse as an individual (Manouze et al., 2019). Given the existing individual variability, averaging values for a given observable may blur the reality. Although more time consuming, we argue that an individual approach may allow the extraction of categories, which may bear translational values (e.g., responders vs non-responders to a treatment).

Each model only revealed the tip of the iceberg. Many important issues remain unaddressed. Perhaps the most important one is determining the seizure onset zone(s) and the propagation pattern in the brain. This requires multi-site recordings, which are regularly done in patients during presurgical evaluation. Such a study has been performed in the pilocarpine model (Toyoda et al., 2015) and the intrahippocampal kainate model (Sheybani et al., 2018). The same approach should now be used in the various KA models. More generally, we argue that the phenotype of each animal model is far from being completely described. Considerable work is needed regarding the type of seizures expressed, their time evolution, their site of origin, their zone of propagation, the morpho-functional reorganizations in different brain regions, and comorbidities (depression, anxiety, cognitive deficits, etc.). Another pressing question is sex. As mentioned, the number of studies performed in males far outweigh those done in females. It is like half of the field is missing. Although more work is done during development and aging, more work is also clearly needed as the mechanisms are likely to be age dependent. Finally, recent studies highlight the necessity to consider circadian and multidien rhythms, the existence of different classes of seizures, and the impact of housing (single or colony), which can add the confounding factor of stress. Perhaps accepting the diversity of the different models and experimental conditions has to offer is the best way to understand human phenotypes’ diversity.

## Synthesis

Reviewing Editor: Katalin Toth, University of Ottawa Decisions are customarily a result of the Reviewing Editor and the peer reviewers coming together and discussing their recommendations until a consensus is reached. When revisions are invited, a fact-based synthesis statement explaining their decision and outlining what is needed to prepare a revision will be listed below. The following reviewer(s) agreed to reveal their identity: NONE.

This review compares various methods of KA administration. The review will be useful for the scientific community given the large number and sometimes contradictory results available in the literature. Identifying the right model, that best fits the purpose of investigating various aspects of seizures and the development of temporal lobe epilepsy is critical, and the current review could lead to better strategies. Both reviewers provided several constrictive comments that could help to improve this review. For example, the reviewers comments can guide the authors to elaborate better how these various models relate to the human condition and what could be behind the observed lab-to-lab differences.

Rev. #1.

Treatment with kainic acid induces in rodents a pattern of neuronal death and delayed emergence of recurring hippocampal seizures similar to the human syndrome of mesial temporal lobe epilepsy. This review compares different methods of KA administration (systemic, intra-ventricular, intra-hippocampal, intra-amygdala and intranasal injection...) and resulting syndromes. While it states "experimental models can provide hypotheses and clues relevant to humans", there is little comparison of morphological damage and circuit re-arrangement or patterns of paroxysmal activities in these models with human data. Neither do the authors conclude which protocol offers the most useful model, although they stress that female animals should be studied in greater detail. Some specific comments are listed below. Overall, the authors do not generate a compelling motivation for the review, even though they have clearly worked hard on it.

1. line 33. ‘comorbidities’. The text lists ‘cognitive deficits, anxiety and depression’ as co-morbidities. But they may be consequent on neuronal death, circuit re-arrangements or seizures induced by KA. Possibly it’s a poor understanding on my part, but I thought that co-morbidity referred to a pre-existing, independent condition that could aggravate a different disease. Such as high blood pressure or diabetes in people infected with the covid-virus. There may be confusion on this definition so an alternative form of words should be found.

2. lines 56-135. I guess its worth to mention pilocarpine, tetanus toxin, kindling and brain injury. If so, the review must ask why they sometimes do or do not induce similar patterns of cell death, axonal sprouting and delayed epileptic activity in the hippocampus.

3. lines 152-181. Even if data presented on kainate receptors is quite complete, the review avoids to conclude which receptor subtypes underly neuronal death or seizure like activity. Are they parts of the same process? How can the ‘unique effect in the hippocampus’ be explained?

4. line 197. ‘plays a central role in cell death’.

5. 241-270. One interesting protocol might be to terminate SE immediately. Do recurrent seizures emerge with a delay after KA-induced neuronal death when status epilepticus does not occur?

6. line 273-283. Why is the latent period so much shorter in rodents than in humans?

7. lines 285-304 and 524-575. Spontaneous recurrent seizures. It seems a sad omission to compare seizures generated in rodents with different modes of KA-treatment but not with the human syndrome. It is true that seizure frequency, clinical signs, the form and duration of discharges and patterns of spread of seizure activity are very variable in MTLE patients. Even so, an explicit comparison would help validate the model.

8. Lines 307-324 Histology for each mode of administration. This format, where patterns of neuronal damage are described separately for each different mode of KA treatment, precludes comparison of similarities or differences. A short section of conclusions that emerge from these comparisons would be useful and should be added.

9. lines 644-662. Useful to raise the question of female vs male animals as well as the strain related variation.

10. lines 669-673. These couple sentences should be eliminated or reworked since they are inconsistent. The introduction states ‘experimental models can provide hypotheses and clues relevant to humans’. Here the text suggests ‘whether or not the KA models are mimicking human TLE is not a relevant question’. It seems impossible to reconcile these statements. If KA models provide no clues relevant to humans, then conclusions are no more, or less, useful or interesting than behaviors and neuronal mechanisms of rhythms generated in the crayfish stomatogastric ganglion.

Rev. #2.

To be transparent, I saw the authors listed on a title page. I thought I would do the review anyway because it is late and I also wanted to provide input. However, I want to make my knowledge of the authors clear so the review can be considered with this in mind. I can allow my name to be stated so that both parties, authors and reviewer, are fully transparent to each other. I apologize not reading the rules earlier so I could notify the Editorial office.

This manuscript is a review about the use of kainic acid (KA) to induce epilepsy in rats and mice. The authors provide a view of the past literature, which is an immense task. There also is unfortunately a lot of data which is not quantitative in the past, especially in the early years when EEG was unavailable, and no one used mice. As the field has evolved, reviews because difficult because it is very hard to pull the disparate literature together.

The result is a solid job, but there are some aspects of the review that could be improved.

Major concerns

In several instances rat and mouse data are discussed together and these are very different. In addition, use of rats without EEG led to a view of chronic seizures that probably was inaccurate. However, not having EEG was only one issue. Laboratories sometimes only quantified for 8 hrs of every 24 or less than 2 weeks which is really the minimum used today. Therefore, separating the summary of rat and mouse work is probably needed.

There are other difficulties with pooling data from different studies that need to be pointed out. Each lab does not really define a seizure the same way. One might think that saying the seizure was stage 5 should make that problem go away, but a stage 5 seizure without EEG can miss other seizures. Also, mice do not always fit the Racine scale.

There are other reasons for variability from lab to lab that are hard to be sure of. These have to do with breeding methods, and things like social housing which is mentioned by the authors. This makes it very hard to make summary statements. However, if these difficulties are acknowledged, then the authors can provide what they think is the best consensus statement.

It should be pointed out that many investigators question whether KA in mice leads to epilepsy in mice. Initial attempts using systemic injection failed to lead to robust chronic seizures, probably because of limited neuronal damage (at least according to early studies, but this was not very comprehensive in its assessment, which was done by labs like Schawecker). Later studies did find some damage using strains other than C57bl6 mice, but here there is another issue - not all C57bl6 mice are the same, even within one country (e.g. the U.S.).

After the use of intrahippocampal, intraamygdala, icv and other types of KA injection in mice began, there were some successes but most papers do not shown convincing seizures. Rather, there are spikes and bursts, usually so frequent it is hard to believe they are all seizures. We found this initially too and then modified the methods to get discrete 30-50 sec convulsive seizures with interseizure intervals >1 hr and sometimes >1 day.

Now there are some investigators who probably have this type of model producing regular convulsive seizures and the authors may be one such laboratory, citing 8 seizures per day which is about what we get in our group after modifying the way we inject. But some investigators have to wait many months for convulsive seizures. Others had found the model does not produce any seizures although SE occurs initially. Still others find seizures are so variable it is no wonder many find few seizures most of the time.

In light of this I suggest the paper be revised to reflect the difficult state of affairs in the field and perhaps with this stated, the authors can then provide their summary and personal experience. That type of review might be considered a perspective rather than a comprehensive review. Using the term perspective rather than comprehensive also allows the authors to provide their opinions, which they do several times in the paper. It takes them ‘off the hook’ in attempting to adequately summarize the very difficult literature.

Neuroanatomy/pathology/histology

First, the authors should clarify what they mean by neuropathology vs histology vs anatomy. Or use one term. On Line 5 neuropath and histology are mentioned together and it is not clear what the authors mean with each term. Second and more importantly, there is a difficulty with the figure and the field. Hippocampal damage can not really be assessed with one or two sections because the damage is so variable when KA is injected into one hippocampus. One also needs to know what occurred on the contralateral side, which is extensively changed in many cases. Then there are dorsal/ventral differences in all the models, leading to very different pathology and compensatory rewiring. While daunting, I think the authors can explain these issues make it difficult to summarize, while still providing their perspective afterwards. Again, by saying it is their perspective the authors are free to summarize the field as they see it.

In light of this, the Figures covering neuropathology, the Klee et al. study, and the Williams et al. study provide great examples of what has been found, but they do not necessarily reflect what all investigators find. That should be stated at some point, and I apologize if I missed it.

Minor comments

Line 7. It is very hard in a review like this to also do justice to the other models. There is just too much to say to do justice to the field. TBI is a good example. This is quite a contentious group of models, with some investigators finding quite convincing chronic seizures and others not so. Despite this several investigators belittle the models that use SE and instead suggest the TBI models are the real models of mesial temporal lobe epilepsy (TLE). The reason is some clinicians do not see a lot of SE in the history of those people who get TLE but do see brain injury. However, other clinicians see SE as quite common. There are many ways of doing TBI as well. The same is true of several of the other models. It might be best to omit this from the review because it’s not going to be possible to do something brief, in my opinion. One possibility as an alternative is a table with numerous references so all the discussion and variations of the models are included.

Line 23. An important part of the definition of TLE is that the seizures come from the medial temporal lobe. That is very important to point out because most of our rodent models do not prove that the seizures come from the hippocampus or other parts of the rodent brain that one would say are analogous to the human medial temporal lobe.

Line 33. I think it would be better to say it is debateable or contentious rather than inadequately addressed so that the authors of the studies addressing the issue are not insulted unnecessarily. In truth, we are all doing the best we can with limited time and resources.

An interesting omission is seizure-induced neurogenesis. This has become a very large field and it is very interesting in the IHKA model because the neurogenesis is massively reduced in the hippocampus ipsilateral to the injection, which Fritschy showed. This is a sharp contrast to the very large increase in neurogenesis in the systemic models and other focal models.

Lines 45-46. There are other models that are not included here and it would make sense to do so, like the continuous hippocampal stimulation of Lothman/Bertram and also continuous perforant path stimulation of Wasterlain’s group.

Line 58. Note there are several genetic disorders and transgenic mice that exhibit a TLE-like phenotype. This fits with the view that TLE has a genetic component and early childhood is often important.

Line 69. I don’t think one can make conclusions about how rapidly damage develops or how rapidly mice exit the latent period (if there is one) because the labs and assays are almost always different in how they do the work and time points assessed. Often damage is assessed without unbiased stereology or the gold standard, fluorojade.

Also it is hard to equate Lithium-Pilocarpine with Pilocarpine with less mortality. The pretreatment really creates a different model. However, trying to provide references for this is definitely hard. So perhaps the best thing to do is state this a bit more cautiously.

Lines 80-83. have a lot of content and the sentence is hard to follow. Breaking this up into more than one sentence would be a solution.

Line 86. Mckhann et al. and Schawecker’s work could be cited here.

Line 84. Systemic KA in rats can have extremely high mortality. Initially that was the case because it was considered only possible to get chronic epilepsy if the anticonvulsants were absent after SE began. Then people began to show less mortality with a post-SE anticonvulsant and there still was robust epilepsy. This greatly changed all SE models, and many people don’t realize that.

Line 103. It is very important that most kindling models fail to show chronic seizures so it is really a model of epileptogenesis but not epilepsy. On the other hand, those who kindle about 100x (Sutula’s group) do see chronic seizures. Also, the continuous hippocampal stimulation model produces SE and it definitely leads to chronic seizures. (Note that CHS is sometimes called self-sustaining SE so it is confusing in the literature.)

Line 197-201. I think the literature actually shows a lot of evidence for both excitotoxic and apoptotic cell death and this is still hotly debated because some believe the assays used for one are poor so the evidence is bad. Yet the proponents of those assays argue the other ones are bad, etc. The field never really resolved the issue and as time elapsed many new investigators who did not know the history only studied apoptosis, leading some to believe that apoptosis is the majority of the reason for cell death. I still don’t think we really know, so both excitotoxicity and apoptosis should be discussed in a balanced way.

Line 226. I’m not sure of this section because many IHKA models don’t show evidence of chronic seizures and many find systemic KA injection has high mortality.

Line 236. I would mention the two forms, the free acid and the dihydrate. We also see the lot to lot variability, and with pilocarpine this could be true, but we don’t notice it as much. Nevertheless, we see different batches of mice even from the same breeding colony vastly differ. We breed in-house so we have more control, and it still occurs. I think many investigators think time of year is an issue and that might be good to mention.

Line 240. The section starting on this line is a good example on the pooling of rat and mouse studies where they are best separated.

Line 262. SE is not "terminated" - it is decreased in severity. We know that from EEG and for that reason "perfect" control should also be reworded.

Line 279. This is an example of discussion of the latent period where I think cautious wording is needed. Again, some of the studies made assumptions about the latent period based on behavioral seizures only (instead of EEG) and may not have studied every day after SE until the first convulsive seizure. So the assessment was not precise. When EEG was used it may only have been a hippocampal electrode and then it is not clear extrahippocampal seizures might have occurred and the investigators were unaware. Also some individuals consider a convulsive seizure to be stage 2, whereas other consider it to be stages 4-5.

I think the discussion of Age is a bit limited. For Age, there is a lot to say about models of childhood insults that lead to TLE, or immature rodents and TLE. The authors might refer the reader to a few good reviews.

Line 304. Note that many investigators cannot house animals together because of the implant used to record EEG. The rats groom each other and the implant usually is damaged.

Line 525. This is another example where pooling rat and mouse data needs to be done cautiously if at all.

Line 536. This line starts a section where there is a lot of opinion, so it is one of the places in the paper that made me suggest the word "perspective" is better for this review than "comprehensive."

Figure 1 shows beautiful schematics. If they are from another paper that reference should be cited in the Legend.

Tables. I would reconsider the references. Some of the items in the table seem to have a lot more references that apply to them than are listed, or the references are only in rats or only in mice, leaving the field a bit incomplete. The gaps in the field are actually good to point out so young investigators see the opportunities that exist for important work. In fact that might be stated as such, because most of the reviews like this just present a confusing picture, leading to disillusionment rather than insight into what the field really needs.
